# Metagenomic Insight into the Microbiome and Virome Associated with *Aedes aegypti* Mosquitoes in Manado (North Sulawesi, Indonesia)

**DOI:** 10.3390/idr15050054

**Published:** 2023-09-11

**Authors:** Janno Berty Bradly Bernadus, Jantje Pelealu, Grace Debbie Kandou, Arthur Gehart Pinaria, Juliet Merry Eva Mamahit, Trina Ekawati Tallei

**Affiliations:** 1 Entomology Study Program, Postgraduate Program, Sam Ratulangi University, Manado 95115, North Sulawesi, Indonesia; jantje_pelealu@unsrat.ac.id (J.P.); grace.kandou@unsrat.ac.id (G.D.K.); arthur.pinaria@unsrat.ac.id (A.G.P.); evamamahit@unsrat.ac.id (J.M.E.M.); 2 Department of Parasitology, Faculty of Medicine, Sam Ratulangi University, Manado 95115, North Sulawesi, Indonesia; 3 Biomolecular Laboratory, Sam Ratulangi University, Manado 95115, North Sulawesi, Indonesia; 4 Faculty of Agriculture, Sam Ratulangi University, Manado 95115, North Sulawesi, Indonesia; 5 Faculty of Public Health, Sam Ratulangi University, Manado 95115, North Sulawesi, Indonesia; 6 Department of Biology, Faculty of Mathematics and Natural Sciences, Sam Ratulangi University, Manado 95115, North Sulawesi, Indonesia

**Keywords:** microbial diversity, metagenomic analysis, whole-genome shotgun sequencing, *Aedes aegypti*, viral diversity

## Abstract

The aim of this study was to investigate the microbial diversity encompassing bacteria, fungi, and viruses within the composite microbial community associated with *Aedes aegypti* mosquitoes in Manado, Indonesia, using a whole-genome shotgun metagenomics approach. Female mosquitoes were collected and grouped into pools of 50 individuals, from which genomic DNA (gDNA) and RNA were extracted separately. Whole-genome shotgun metagenomics were performed on gDNA samples. The bioinformatics analysis encompassed quality assessment, taxonomic classification, and visualization. The evaluation of the microbial community entailed an assessment of taxa abundance and diversity using Kraken version 2.1.2. The study delineated the prevalence of dominant bacterial phyla, including Proteobacteria, with varying abundance of Firmicutes, Bacteroidota, and Actinobacteria, and notable occurrence of Tenericutes. Furthermore, the presence of the fungal phylum Ascomycota was also detected. Among the identified barcodes, Barcode04 emerged as the most abundant and diverse, while Barcode06 exhibited greater evenness. Barcode03, 05, and 07 displayed moderate richness and diversity. Through an analysis of the relative abundance, a spectrum of viruses within *Ae. aegypti* populations was unveiled, with Negarnaviricota constituting the most prevalent phylum, followed by Nucleocytoviricota, Uroviricota, Artverviricota, Kitrinoviricota, Peploviricota, Phixviricota, and Cossaviricota. The presence of Negarnaviricota viruses raises pertinent public health concerns. The presence of other viral phyla underscores the intricate nature of virus–mosquito interactions. The analysis of viral diversity provides valuable insights into the range of viruses carried by *Ae. aegypti*. The community exhibits low biodiversity, with a few dominant species significantly influencing its composition. This has implications for healthcare and ecological management, potentially simplifying control measures but also posing risks if the dominant species are harmful. This study enriches our comprehension of the microbiome and virome associated with *Ae. aegypti* mosquitoes, emphasizing the importance of further research to fully comprehend their ecological significance and impact on public health. The findings shed light on the microbial ecology of *Ae. aegypti*, offering potential insights into mosquito biology, disease transmission, and strategies for vector control. Future studies should endeavor to establish specific associations with *Ae. aegypti*, elucidate the functional roles of the identified microbial and viral species, and investigate their ecological implications.

## 1. Introduction

*Aedes aegypti* is a mosquito species known for its role in transmitting several diseases, including dengue fever, Zika, and chikungunya [1,2]. These diseases pose significant public health challenges in many parts of the world [3]. *Aedes aegypti* mosquitoes are particularly adept at transmitting these pathogens, making them a major concern for disease control efforts [4].

Understanding the microbiome and virome associated with *Ae. aegypti* mosquitoes is essential due to the significant public health challenges posed by the diseases they transmit. Knowledge about the mosquito’s disease transmission capability and potential targets for disease control strategies can be obtained through the examination of the microbiome and virome [5,6]. Interactions between the mosquito and its microbiome influence various aspects of mosquito biology, such as reproduction and immune responses [7]. In addition, studying the virome permits the identification and characterization of the numerous viruses present in *Ae. aegypti* mosquitoes, thereby enhancing our understanding of the viral landscape and assisting in the prediction and monitoring of disease outbreaks. The virome analysis also provides insights into the potential impact of viruses on disease transmission and the interactions between mosquitoes and pathogens [8,9,10,11,12]. Through a comprehensive investigation of the microbiome and virome, novel strategies can be developed to disrupt disease transmission cycles and alleviate the public health consequences associated with *Ae. aegypti* mosquitoes.

During 2022, a substantial increase was observed in the incidence of dengue fever infections within North Sulawesi, Indonesia. The documented cases surged from 135 in the previous year to 598, reflecting a significant escalation. Over the span of January to June 2023, a cumulative count of 1120 cases were recorded. Notably, Manado emerged as the area with the highest caseload, comprising 230 cases. The observed rise in cases was particularly significant in the Malalayang I Subdistrict and Bahu Subdistrict, both located within the Malalayang District. A combined total of 122 cases were reported in these areas. On the other hand, the Paal 2 region documented a limited number of cases, specifically six.

The Malalayang Subdistrict is located in a coastal region and possesses a unique environmental and ecological landscape. This landscape is characterized by residential areas that are specifically designated for the local community, as well as a notable degree of population mobility. The region also enjoys the advantages of a strong healthcare infrastructure, which encompasses hospitals and community health centers. Additionally, there is a wide array of educational facilities available, ranging from primary schools to tertiary institutions. On the other hand, the Paal 2 Subdistrict exhibits a comparatively lower population density and is geographically positioned farther from coastal regions and healthcare amenities. The level of human activity in Paal 2 is significantly lower in comparison to that observed in the Malalayang Subdistrict. The recent increase in the number of cases, coupled with the limited amount of data on the existence of pathogenic microorganisms (such as bacteria and viruses) within *Ae. aegypti* mosquitoes, which are recognized carriers of the disease, has sparked the interest of researchers. Taking advantage of the progress made in molecular sequencing techniques, a metagenomic study was initiated to elucidate the genomic information related to bacteria and viruses present in *Ae. aegypti* in Manado.

The primary aim of this study involved investigating the microbial and viral communities that are associated with populations of *Ae. aegypti* mosquitoes in Manado, Indonesia. To achieve this aim, a total of 50 mosquitoes were grouped together in each sample (designated as a “barcode”). The present study employed a thorough examination of the entire genome of the metagenome to investigate the proportions and diversity of microbial and viral populations within the mosquitoes. The findings of this study are anticipated to yield substantial implications for enhancing our understanding of mosquito microbiomes and viromes, thus providing insight into the complex interactions between mosquitoes, bacteria, and viruses. The knowledge obtained from this research has the potential to serve as a foundation for the development of innovative strategies aimed at mosquito control and disease prevention.

## 2. Materials and Methods

### 2.1. Sampling Locations

**Figure 1:**
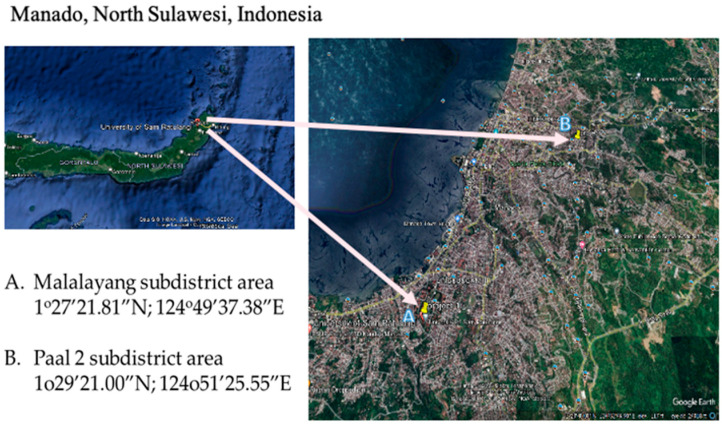
The geographical locations and the coordinates of mosquito sampling sites. Location A is situated in the Malalayang region, while Location B is situated in the Paal 2 area. The distance between these two locations is approximately 10 km.

The mosquito specimens were captured in two specific areas of Manado City, North Sulawesi, Indonesia, namely the Malalayang area (1°27′21.81″ N 124°49′37.38″ E) and the Paal 2 area (1°29′21.00″ N 124°51′25.55″ E). The geographical locations of these areas are depicted in Figure 1. Manado, situated in North Sulawesi, Indonesia, undergoes two distinct seasons: a wet season and a dry season. Throughout the wet season, extending from November to March, Manado encounters recurrent rainfall and intermittent thunderstorms. This temporal phase is marked by heightened humidity levels. In contrast, the dry season spans from April to October. Within this season, there is a decline in precipitation, culminating in comparatively arid and steady weather conditions.

### 2.2. Sample Preparation

Mosquitoes were collected using light traps and mosquito nets during the day. The collected mosquito samples were initially placed in a cup with a net and then transferred to an icebox for transportation to the laboratory. The collection of mosquitoes was conducted over a two-month period from February to March 2023. A team of 15 individuals utilized entomological sweeping nets for the capture sessions, which were strategically scheduled to take place in the morning between 07:00 and 10:00 a.m., as well as in the afternoon from 03:00 to 05:00 p.m.

In the laboratory, the morphological identification of the samples was conducted using a stereomicroscope with a 400× magnification, and photographic records were captured at 4 K resolution. The morphology identification keys were specifically designed for *Aedes* mosquito species, as provided by the Ministry of Health of the Republic of Indonesia. Female mosquitoes were identified and subsequently aggregated into pools of 50 specimens per microtube. Each microtube was assigned a unique barcode identifier. The samples were further divided into two groups for subsequent analysis: the first group was treated with 1 mL of nuclease-free water in a tube for DNA extraction, while the second group was treated with phosphate-buffered saline (PBS) for RNA extraction. After preparation, the specimens were stored at −80 °C until DNA and RNA extraction.

#### 2.2.1. gDNA and RNA Extraction

The mosquitoes were mechanically disrupted using a pestle grinder instrument within a microtube containing nuclease-free water for DNA preservation and phosphate-buffered saline (PBS) for RNA preservation. Subsequently, the samples underwent extraction using a Qiagen DNA Mini Kit to isolate genomic DNA (gDNA) and a Qiagen RNA Virus Mini Kit to isolate RNA. Additionally, the quantity of gDNA was assessed using a Qubit fluorometer (Thermo Scientific, Wilmington, DE, USA) to ensure it met the required threshold for subsequent analyses.

#### 2.2.2. Whole-Genome Shotgun Metagenomics (Wf-Metagenomics)

The extraction procedures yielded six pooled DNA samples and two pooled RNA samples, which were suitable for subsequent wf-metagenomic analysis. The DNA samples designated for wf-metagenomics were given codes from Barcode01 to Barcode06, while the RNA samples were coded as P1 and P2.

The genomic DNA (gDNA) samples underwent processing to generate the metagenomic amplicon library, following the Gridion MK-1C Oxford Nanopore Technology protocol. For the amplification of the amplicons, the Rapid PCR-barcoding kit (SQK-RPB114.24) was employed. This kit includes a set of 24 primers specifically designed to amplify each individual sample. Each primer consists of a unique barcode and a 5’ tag that facilitates the attachment of rapid sequencing adapters, thereby eliminating the need for ligation. Following the amplification and barcoding procedure, the samples were pooled together, and rapid sequencing adapters were introduced to the combined mixture. To initiate the sequencing process on the Gridion MK-1C Oxford Nanopore Technology device, the Flow Cell Priming Kit (EXP-FLP002) was utilized. Furthermore, the gDNA samples were also prepared for the virome metabarcoding process amplicon library, following the same Gridion MK-1C Oxford Nanopore Technology protocol. For sequencing the amplicons in this case, the cDNA-PCR Sequencing kit (SQK-PCS111) was employed.

#### 2.2.3. Bioinformatics Analysis

The following protocol described the steps for analyzing Gridion MK-1C Oxford Nanopore sequencing results using EPI2ME version 5.1.2 for metagenomic bioinformatics analysis [13,14]. The raw sequencing data were retrieved from the Gridion MK-1C sequencer and converted to FASTQ format. Subsequently, the FASTQ files were uploaded to the EPI2ME version 5.1.2, and the “Metagenomic Analysis” workflow was selected. Parameters such as read quality and adapter trimming were set accordingly. The analysis was then initiated, and the progress was monitored through the EPI2ME version 5.1.2 interface. Upon completion of the analysis, the results were reviewed and exported to a designated location for further analysis.

In order to enhance the analytical process, the sequencing reads underwent alignment against either a reference genome or a pertinent database by using the Minimap2 tool. The resulting SAM or BAM files were collected for downstream analysis. The aligned reads were subjected to taxonomic classification based on pertinent taxonomic information using Kraken version 2.1.2 [15]. This procedure generated a comprehensive report or output file detailing the results of the taxonomic classification. The taxonomic classification results derived from Kraken were used to generate Krona visualization [16]. These visual representations provided insights into the relative abundance of different taxonomic groups within the metagenomic samples. The combined results obtained from EPI2ME, Minimap2, Kraken, and Krona were then analyzed and interpreted, facilitating the comprehension of the metagenomic profile inherent to the samples.

#### 2.2.4. Microbial Community Analysis

Relative abundance was obtained from taxonomic annotations using TaxonKit [17]. This information was visualized through the utilization of Kraken, which enables the creation of charts at both the phylum and genus/species levels. These charts allow for a visual representation of the most abundant taxa and their proportions within the sample’s classification level. The analysis conducted with Krona facilitates the generation of these visualizations, providing a clear and intuitive comprehension of the composition and distribution of taxa in the metagenomic samples.

#### 2.2.5. Alpha Diversity Analysis

Alpha bacterial diversity within *Ae. aegypti* was quantified using Kraken. The taxonomic composition of the bacterial community associated with *Ae. aegypti* was assessed and measures of diversity were calculated using the Simpson (1-D) and Shannon–Wiener (H’) indices. The Simpson (1-D) index indicated the richness and evenness of bacterial taxa distribution, while the Shannon–Wiener (H’) index provided a comprehensive assessment of diversity by considering both species richness and relative abundances.

## 3. Results and Discussion

Wf-metagenomics can detect both bacteria and fungi simultaneously in a mixed microbial community. Since wf-metagenomics involves sequencing the entire genomic DNA extracted from a sample, it captures the genetic material from all organisms present, including bacteria, fungi, archaea, viruses, and other microorganisms [18]. In the present study, a total of 600 female mosquitoes were collected: 350 from the Malalayang area and 250 from the Paal 2 area. These mosquitoes were grouped into pools of 50 individuals per tube, resulting in eight DNA extraction pools—five from the Malalayang area and three from the Paal 2 area. Additionally, 200 mosquitoes were allocated for RNA extraction and were divided into two pools for each of the Malalayang and Paal 2 areas. Subsequent quantification of DNA and RNA concentrations, conducted using a Qubit fluorometer, indicated that only six of the DNA pools and two of the RNA pools satisfied the criteria necessary for subsequent library preparation for metagenomic sequencing. Pools designated for metagenomic analysis were assigned unique barcode identifiers, whereas those intended for viral metabarcoding were labeled with a “P” prefix. Specifically, Barcode03, Barcode04, Barcode05, Barcode06, Barcode07, and P1 were sourced from the Malalayang area, while Barcode08 and P2 originated from the Paal 2 region.

### 3.1. Microbial Relative Abundance

**Figure 2:**
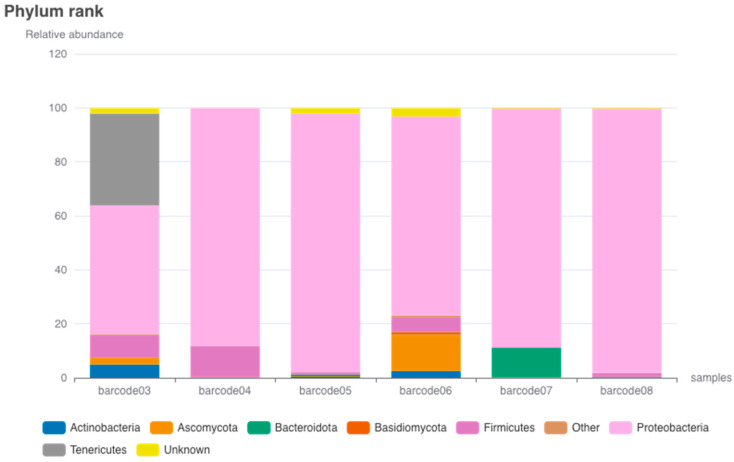
The distribution and relative abundance of bacterial and fungal phyla in *Ae. aegypti* in each sample. The barcodes with identifiers Barcode03, Barcode04, Barcode05, Barcode06, and Barcode07 were acquired from the Malalayang region, whereas Barcode08 was obtained from the Paal 2 region.

The analysis of the barcode data reveals interesting patterns in the distribution of phyla (Figure 2). Furthermore, detailed data regarding the species identified within each specific barcode are presented in Supplementary Figure S1, which displays the corresponding phylogenetic diagrams. Simultaneously, Supplementary Figure S2 presents a Krona visualization to provide additional analytical context. Proteobacteria emerges as the most dominant phylum, exhibiting high percentages ranging from 47.73% in Barcode03 to an impressive 97.65% in Barcode08, making it the predominant phylum across all barcodes. In contrast, Firmicutes displays varying presence among the barcodes, with the highest percentage of 11.54% in Barcode04 and the lowest percentage of 0.45% in Barcode07. Firmicutes is generally less abundant compared to Proteobacteria.

Bacteroidota, on the other hand, exhibits a relatively low representation, with its highest percentage of 10.83% in Barcode07 and the lowest percentage of 0.04% in Barcode05. Actinobacteria, while relatively low in abundance, shows a peak percentage of 4.91% in Barcode03, declining in the other barcodes. Actinobacteria is not as prevalent as Proteobacteria. The presence of Bacteroidota is not consistent across the barcodes. These findings align with previous research, which demonstrated that the phylum Proteobacteria was the dominant group in the midgut microbiota of *Ae. albopictus* and *Ae. aegypti* from Arunachal Pradesh, India. Subsequently, the phyla Firmicutes, Bacteroidetes, and Actinobacteria were also observed in significant abundance [19]. Furthermore, it has been documented that in a population of *Ae. albopictus* originating from Réunion Island in France, bacterial communities at the phylum level were primarily dominated by Proteobacteria. Bacteroidetes, Actinobacteria, and Firmicutes followed as the subsequent prevalent taxa, irrespective of the specific organ or sex [19].

Tenericutes exhibits notable prominence within Barcode03, constituting a substantial proportion of 34.09%, whereas its occurrence in other barcodes is almost negligible. This finding implies a distinct association between Tenericutes and Barcode03. However, in a previous study, Tenericutes was identified to account for less than 4% of the microbial composition in *Ae. aegypti* from Colombia [20]. The observed patterns of microbial abundance and diversity are likely influenced by a combination of host-specific factors, environmental conditions, and ecological interactions.

Ascomycota, a phylum of fungi, demonstrates varying percentages across the barcodes, with the highest percentage of 13.44% in Barcode06 and the lowest percentage of 0.06% in Barcode08. Ascomycota generally appears in smaller proportions compared to other phyla. The phylum labeled as “Unknown” exhibits a moderate presence in most barcodes, ranging from 0.06% to 3.26%. The “Unknown” label indicates that the phylum of these organisms has not been identified or categorized. The remaining phyla, including Basidiomycota, Zoopagomycota, Cyanobacteria, Deinococcus-Thermus, Mucoromycota, Chytridiomycota, Crenarchaeota, Chloroflexi, and Spirochaetes, generally exhibit low percentages across all barcodes, indicating their minor presence.

Among a group of *Ae. albopictus* derived from Réunion Island in France, the phylum Ascomycota exhibited the highest prevalence, closely followed by Basidiomycota. In female mosquitoes, the families Weeksellaceae and Burkholderiaceae were identified as the most abundant in crops (21.2%) and guts (14.3%), respectively [21]. In a previous study, a comparative analysis was performed between three indigenous populations of *Ae. albopictus* in Vietnam and six populations of *Ae. albopictus* recently introduced in France and Madagascar. The study findings unveiled that all mosquito populations harbored a structured fungal community, characterized by a significant prevalence of yeasts within their “core mycobiota.” Nonetheless, the invasive populations from France and Madagascar exhibited a notable reduction in fungal diversity in contrast to the native populations in Vietnam [22].

The notable presence of Ascomycota at a relatively high percentage in Barcode06 suggests its significant contribution to the microbial composition within that specific sample. This observation implies that environmental factors or ecological interactions might favor the growth and proliferation of Ascomycota in Barcode06. Ascomycota, being a diverse and widely distributed phylum encompassing various fungi, exhibits variations in its percentages across the barcodes. These variations may reflect the inherent diversity and adaptability of Ascomycota to different environmental conditions. Factors such as substrate availability, moisture content, pH levels, and interactions with other microorganisms can influence the relative abundance of Ascomycota in a given sample. Ascomycota represents the most extensive fungal group, known for its remarkable ecological diversity, comparable to the pathogenicity exhibited by this group towards plants, animals, and humans. Within the Ascomycota, there exists a substantial collection of pathogenic insect fungi, which display a broad spectrum of insect targets, making them the prevalent pathogens among insects [23].

**Figure 3:**
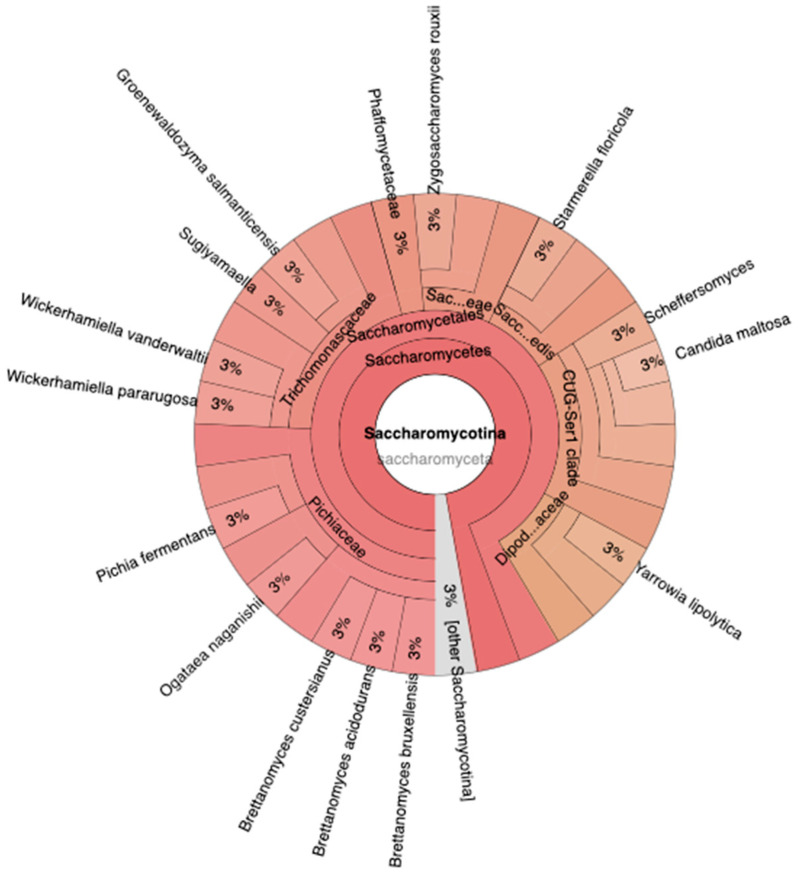
A diverse array of fungal species within the Barcode06 dataset, all falling under the Ascomycota phylum. Each of these species contributes approximately 3% to the overall fungal composition found within the Barcode06 dataset. This near-equivalent representation of these species within Barcode06 suggests a relatively homogeneous distribution.

In the context of Barcode06, the phylum Ascomycota encompasses various fungal species (Figure 3). These fungi include *Wicherhamiella vanderwaltii*, *W. pararugosa*, *Pichia fermentas*, *Ogataea naganishii*, *Breattanomyces cutersianus*, *B. acidodurans*, *B. bruxellensis*, *Yarrowia lipolytica*, *Candida* sp., *Sarmerella floricola*, *Zygosachharomyes rouzii*, and *Groenewaldozyma salmanticensis*. Each of these species constitutes approximately 3% of the total fungal composition within Barcode06. The fact that each of these species accounts for approximately 3% of the fungal composition within Barcode06 suggests that they are relatively evenly distributed. This implies that these species may have comparable abundances and potential ecological importance within the microbial community of Barcode06. However, this information alone does not provide insights into their specific roles, interactions, or functional implications within the ecosystem.

Supplementary Table S1 presents a comprehensive examination of the comparative prevalence of microbial species among distinct barcodes, revealing significant disparities in microbial composition and diversity. Bacterial taxa exhibit widespread representation across various environments, whereas fungi are predominantly restricted to Barcode03 and Barcode07. Significantly, *Asaia bogorensis* in Barcode03 and *Erwinia piriflorinigrans* in Barcode07 exhibit a notable prevalence compared to their counterparts in other barcodes. The observed patterns of species diversity indicate that Barcode07 exhibits the highest level of diversity, followed by Barcode04 and Barcode03. In contrast, Barcode05 and Barcode06 demonstrate relatively lower taxonomic diversity, with a notable emphasis on specific genera such as *Aeromonas* and *Enterobacter*.

The presence of diverse microbial compositions within *Ae. aegypti* mosquito populations has significant implications for comprehending the microbiome. This variability may have an effect on the varying rates of infection observed in different geographical or ecological contexts. Certain strains of *Brettanomyces* found in Barcode07 exhibit distinct characteristics, suggesting that localized microbial interactions may play a role in determining the susceptibility of mosquitoes to pathogens. On the other hand, species that are frequently found serve as fundamental indicators of crucial microbiome characteristics that have a universal impact on interactions between mosquitoes and pathogens. Fluctuations in the prevalence of these ubiquitous species have the potential to influence microbial dynamics at a local level, consequently impacting the vulnerability to infections.

The observed variations in microbial composition and abundance indicate that each barcode potentially corresponds to a distinct ecological niche or different sampling conditions. The observed variations in infection rates may be attributed to a multitude of environmental and ecological factors, such as temperature and humidity, thereby providing additional insight into the disparities observed. A comprehensive comprehension of this subject matter has the potential to improve the efficacy of specific disease control tactics, such as the utilization of microbiome manipulations to mitigate the transmission of pathogens.

The findings from the analysis of the relative abundances within the sample pool have significant implications for understanding the microbial diversity within *Ae. aegypti* and its relation to the host. While the specific connection between the sample pools and *Ae. aegypti* cannot be determined from the given data alone, the broader implications can be discussed. The composition and abundance of phyla within different samples reflect the complexity of the mosquito’s microbiota and its potential influence on mosquito biology, disease transmission, and vector control strategies. The microbial communities represented by the sample pools may impact mosquito physiology, including development, reproduction, immunity, and behavior. Furthermore, the interplay between the mosquito’s microbiota and pathogens, as suggested by specific sample pools, could have implications for vector competence and disease transmission dynamics. Understanding the microbial diversity within *Ae. aegypti* populations, as indicated by the barcodes, can guide the development of targeted interventions and biological control strategies. However, further research and investigations focused on *Ae. aegypti*’s microbiota are necessary to establish the specific associations between the barcodes and the mosquito host. These findings lay the groundwork for exploring the microbial ecology of *Ae. aegypti* and its relevance to mosquito biology and disease transmission.

### 3.2. Microbial Alpha Diversity

Microbial alpha diversity refers to the measurement of species diversity within a specific sample or ecosystem. In the context of *Ae. aegypti*, microbial alpha diversity refers to the diversity of microorganisms, including bacteria and fungi, that are associated with these mosquitoes. Table 1 displays the findings regarding the analysis of microbial diversity in various samples, denoted by Barcode03, 04, 05, 06, 07, and 08. The “Total Counts” column represents the total number of sequencing reads or counts obtained for each barcode. It is worth noting that Barcode04 has the highest total count, indicating its abundance in the sample compared to the other barcodes.

Richness pertains to the count of distinct species or taxa found within a sample. It disregards the abundance or distribution of species in terms of their relative frequencies or proportions. Ecosystems with a high species richness demonstrate enhanced stability, providing them with greater resilience against disturbances [24,25].

The examination of alpha diversity across various sampling groups, specifically from Barcode03 to Barcode08, yielded significant variations as indicated by several metrics. The highest microbial population was observed in Barcode04, with a recorded count of 37,019, which was notably higher than the count of the second-highest group, Barcode08, which had a population of 13,438. In contrast, Barcode03 and Barcode06 exhibited significantly reduced microbial abundances, with values of 2775 and 2485, respectively. In relation to species richness, it is observed that Barcode04 exhibits the greatest diversity, comprising a total of 629 unique species. This finding implies the presence of a complex microbial community with ecological intricacies. On the other hand, the lower level of species richness observed in Barcode03 and Barcode06, comprising 237 and 223 species, respectively, suggests a microbial assembly that is comparatively less diverse.

Further analysis of the Shannon diversity index serves to emphasize these patterns. The Shannon diversity index (H) is a measure used to quantify the overall diversity within a sample, taking into account both species richness and evenness [26]. Barcode08 is identified as the most diverse, exhibiting a Shannon index value of 4.62. Following closely is Barcode04 with a value of 3.94, while Barcode03 demonstrates the lowest diversity with a value of 3.14. In terms of the measure of effective number of species, Barcode04 and Barcode08 exhibit superior performance, with approximate values of 51 and 50, respectively. These values suggest a more equitable representation of microbial species. In contrast, Barcode03 and Barcode05 exhibit lower levels of diversity, as they report values of approximately 23. The effective N species is an estimation of the effective number of equally abundant species that would yield the same diversity as the observed sample [27,28]. Simpson’s index (D) measures the probability that two individuals randomly selected from the sample belong to the same species [29]. To obtain the inverse of Simpson’s index (D), the reciprocal of D is taken. Higher values of the inverse of D represent higher diversity. Both Barcode04 and Barcode06 present high values at 0.93, signifying reduced microbial diversity due to increased species uniformity. Contrarily, Barcode07 registers the lowest value at 0.82, which, in conjunction with the highest inverse of the Simpson’s index at 1.22, suggests a more heterogeneous microbial community. Pielou evenness is a measure that assesses the even distribution of species abundance within a sample, ranging from 0 to 1, where a value of 1 indicates perfect evenness [30]. Pielou evenness values reveal Barcode06 as having the most equitable species distribution with a score of 0.65, while Barcode07 exhibits the least, scoring at 0.56.

The results of this study highlight that Barcode04 and Barcode08 generally demonstrate higher microbial diversity across multiple metrics, whereas Barcode07 exhibits reduced diversity, appearing to be more strongly influenced by a few dominant species. These findings offer valuable insights into the diversity and distribution patterns of microbial species represented by each barcode in the analyzed samples.

### 3.3. Viral Relative Abundance

**Figure 4:**
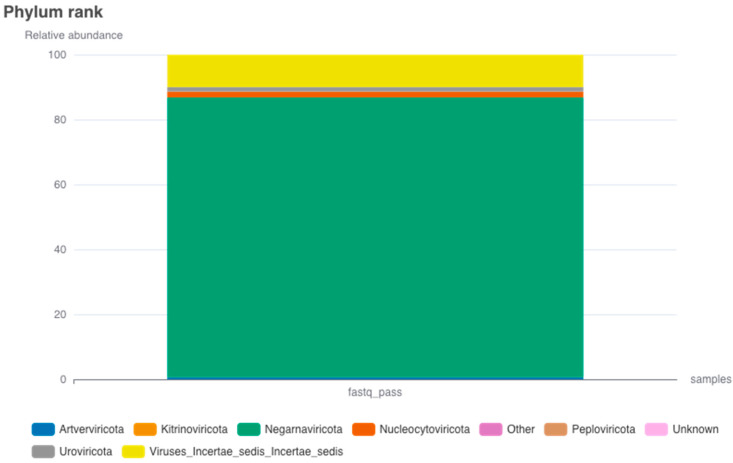
The relative abundance of viral phyla in *Ae. aegypti*, with Negarnaviricota being the most abundant at 8178, followed by Viruses_Incertae_sedis at 951.33. Nucleocytoviricota, Uroviricota, and Artverviricota have moderate to low abundances at 166, 124, and 53, respectively. Kitrinoviricota and Peploviricota are minimally represented with abundances of 8 and 5. The “Unknown” category accounts for 3, while Phixviricota and Cossaviricota are least represented with abundances of 2 and 1, respectively.

The presence of viruses in *Ae. aegypti* holds significant implications for both public health and ecological dynamics. The relative abundance of virus phyla (Figure 4) provides insights into the existence and distribution of these viruses within *Ae. aegypti* populations. The phylum Negarnaviricota is most prevalent, exhibiting a relative abundance of 8178, thereby indicating a significant representation of viruses from this taxonomic group. The category designated as Viruses_Incertae_sedis (virus with uncertain placement) follows with a relative abundance of 951.33. Nucleocytoviricota is moderately represented with a relative abundance of 166, while both Uroviricota and Artverviricota manifest lower abundances, amounting to 124 and 53, respectively. Further, Kitrinoviricota and Peploviricota display even lesser relative abundances, registering at 8 and 5, respectively. The category labeled “Unknown” comprises viruses yet to be classified, and it accounts for a minimal relative abundance of 3. Phixviricota and Cossaviricota have minimal representation in *Ae. aegypti*, with relative abundances of 2 and 1, respectively. In a previous study, the taxonomic phylogeny of 57 known RNA viruses and 39 potentially novel RNA viruses was confirmed through the generation of phylogenetic trees, utilizing the protein sequences of RdRp. The 39 novel RNA viruses were classified into five phyla: Lenarviricota, Pisuviricota, Kitrinoviricota, Duplornaviricota, and Negarnaviricota [31].

The presence of Negarnaviricota viruses in this insect may contribute to the transmission and maintenance of these viral diseases, thereby posing a significant public health concern. The phylum Negarnaviricota was formally established by the International Committee on Taxonomy of Viruses (ICTV) in 2019. This phylum encompasses negative-sense RNA viruses, unified by the common feature of having virally encoded RNA-directed RNA polymerases (RdRps). It is divided into two subphyla: Haploviricotina and Polyploviricotina. Haploviricotina comprises negative-sense RNA viruses that possess RdRps with mRNA capping activity, whereas Polyploviricotina includes those lacking this particular function [32]. To date, no information has been published regarding the presence of Negarnaviricota viruses in *Ae. aegypti*.

The considerable relative abundance of the category Viruses_Incertae_sedis, although lower than that of the phylum Negarnaviricota, necessitates further scrutiny. Investigating the composition and nature of the viruses within this category is crucial not only for understanding their impact on *Ae. aegypti* and the broader ecosystem, but also for devising effective strategies to mitigate potential threats to human health and control mosquito-borne diseases. Furthermore, the presence of other virus phyla, such as Nucleocytoviricota, Uroviricota, Artverviricota, Kitrinoviricota, Peploviricota, Phixviricota, and Cossaviricota, albeit in lower relative abundances, indicates the diversity of viruses coexisting within *Ae. aegypti* populations. While the specific characteristics and implications of these viruses may vary, their presence reinforces the complexity of virus–mosquito interactions and the need for continued surveillance and research.

Studying the existence of viruses in *Ae. aegypti* is crucial for understanding the transmission dynamics of mosquito-borne diseases and developing effective control strategies. By unraveling the interactions between viruses and *Ae. aegypti*, scientists and public health professionals can enhance their ability to predict, prevent, and respond to outbreaks, ultimately safeguarding human populations from the risks associated with these mosquito-borne diseases.

**Figure 5:**
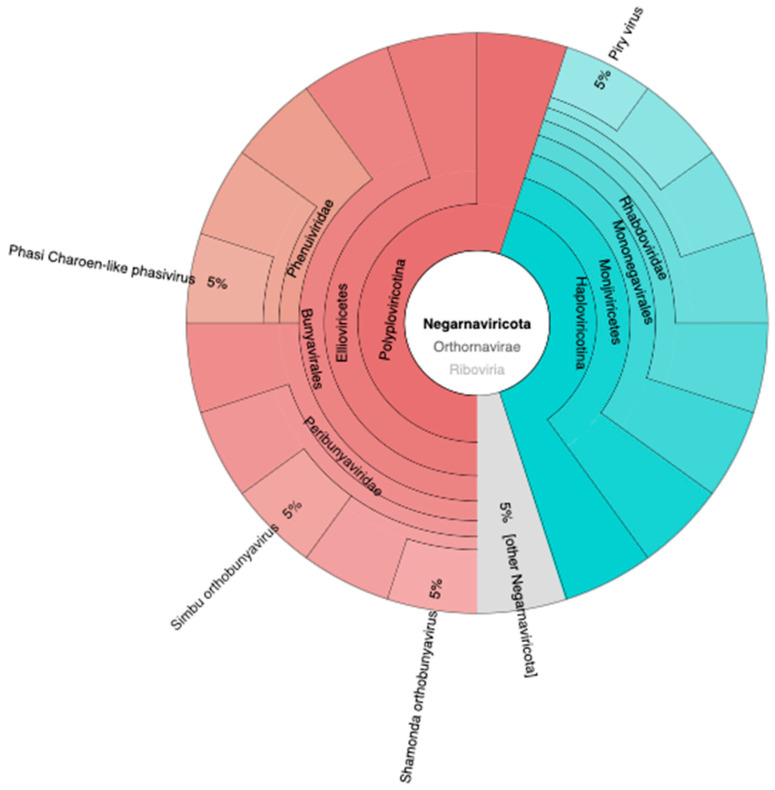
The variety of viruses detected within the taxonomic phylum Negarnaviricota.

Figure 5 illustrates the presence of diverse viruses identified within the taxonomic phylum Negarnaviricota. These include the Phasi Charoen-like phasivirus, accounting for approximately 5% of the identified viruses within this phylum. Similarly, the Piry virus, Simbu orthobunyavirus, and Shamonda orthobunyavirus each constitute approximately 5% of the identified viruses within this phylum. Additionally, there are other viruses belonging to the Negarnaviricota, collectively making up 5% of the identified viruses within this phylum.

**Figure 6:**
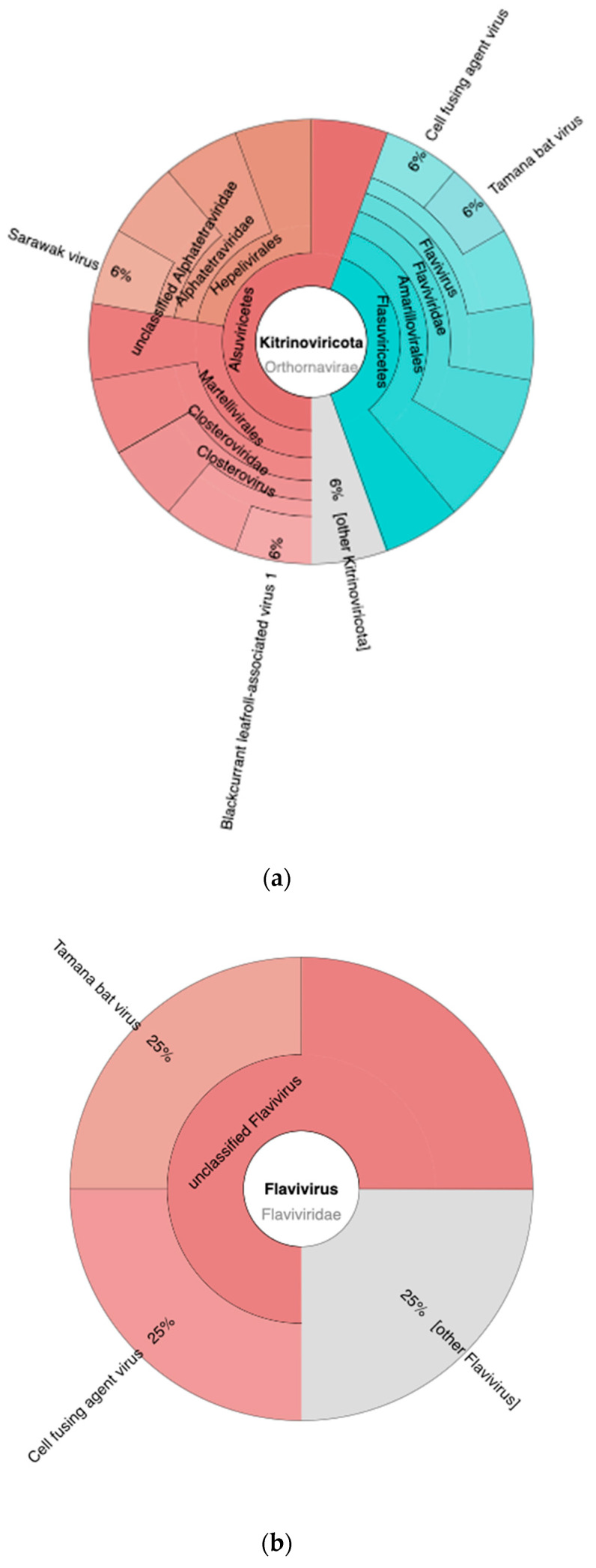
A variety of viruses have been detected within the taxonomic phylum Kitrinoviricota (**a**) and genus Flavirirus (**b**).

Figure 6 illustrates the detection of various viruses within the taxonomic phylum Kitrinoviricota (Figure 6a), which encompasses the genus *Flavivirus* (Figure 6b) as one of its members. Within the phylum Kitrinoviricota, the viruses identified included Sarawak virus (6%), blackcurrant leafroll-associated virus 1 (6%), cell-fusing agent virus (6%), Tamana bat virus (6%), and other viruses belonging to the Kitrinoviricota classification (6%). Within the genus *Flavivirus*, Tamana bat virus (25%), cell-fusing agent virus (25%), and other *Flavivirus* (25%) were detected. The genus *Flavivirus* constitutes a taxonomic assemblage of viruses, comprising 53 defined species, one of which is dengue virus [33]. However, in the current study, the dengue virus was neither detected nor identified; it is plausible that it may be subsumed under other detected *Flavivirus* species.

Flavivirus is a genus within the family Flaviviridae, which is classified under the order Amarillovirales, a part of the class Flasuviricetes. This class falls within the phylum Kitrinoviricota, a group of RNA viruses. Within this taxonomic phylum, the dengue virus, which is the etiological agent for dengue hemorrhagic fever, is present in minuscule quantities. The limited presence of the dengue virus within the sample set has multiple implications of significance. Firstly, it emphasizes the complex nature of the virus and its ability to persist and replicate within the mosquito vector. Despite the low viral load, the virus is still capable of establishing an infection in humans [34,35]. Secondly, the low levels of dengue virus within mosquitoes can impact the efficiency of transmission. Mosquitoes require a sufficient quantity of the virus in the blood to become infected and subsequently transmit the virus to other individuals. With low viral loads, mosquitoes may need to feed on multiple infected individuals in order to acquire enough virus for effective transmission [36]. This aspect of the virus’s transmission dynamics can influence the spread and epidemiology of dengue outbreaks.

### 3.4. Viral Alpha Diversity

Viral alpha diversity in *Ae. aegypti* is important for understanding the variety of viruses they can carry and transmit to humans. It provides insights into viral community dynamics, helps assess disease transmission risks, and aids in designing effective control strategies. Monitoring viral alpha diversity enables early detection of changes in viral populations and facilitates timely surveillance and prevention efforts. Table 2 presents the results of the viral alpha diversity analysis conducted on the samples. The dataset provided includes information on a viral community, with total counts of sequence reads comprising 12,788 for P1 and 14,248 for P2. Within this dataset, there are 66 and 44 unique species in P1 and P2, respectively, indicating the richness of the community. The community’s diversity was evaluated using multiple indices, including a Shannon diversity index of 0.86 for P1 and 0.85 for P2, along with an effective number of 2.37 for P1 and 2.34 for P2. These metrics, along with a Simpson’s index of 0.46 for P1 and 0.51 for P2, and inverse Simpson’s index values of 2.18 for P1 and 1.95 for P2, indicate low diversity and moderate species dominance. In simpler terms, the community is largely influenced by a few dominant species that overshadow less prevalent ones. This pattern is further supported by a Pielou evenness index values of 0.21 for P1 and 0.22 for P2, which highlights an uneven distribution of species abundances. Such low effective diversity and uneven distribution have implications for healthcare and ecological management, particularly for focusing control measures on these dominant types. In contexts like viral or other pathogenic threats, this could simplify management but also pose significant risks if the dominant species are especially harmful.

## 4. Conclusions

Wf-metagenomics in *Ae. aegypti* mosquitoes in Manado, Indonesia, has revealed valuable insights into the microbial and viral diversity in these populations. The analysis identified dominant phyla such as Proteobacteria, Firmicutes, Bacteroidota, and Actinobacteria, as well as the occurrence of Tenericutes and Ascomycota. These findings improve our understanding of the microbial ecology of *Ae. aegypti* and have implications for mosquito biology, disease transmission, and vector control. Further research is needed to establish specific associations with *Ae. aegypti*, explore the functional roles of identified species, and investigate the ecological implications. The analysis of viral alpha diversity highlighted the presence of various viruses in *Ae. aegypti* populations, with Negarnaviricota being the most abundant phylum. The abundance of Negarnaviricota viruses raises public health concerns, while the presence of other virus phyla emphasizes the complexity of virus–mosquito interactions. Monitoring viral alpha diversity is crucial for understanding disease transmission risks and developing effective control strategies.

## Figures and Tables

**Table 1:** Microbial alpha diversity.

Sample Pools	Total Counts	Richness	Shannon Diversity Index (H)	Effective N Species	Simpson’s Index (D)	Inverse of D	Pielou Evenness
Barcode03	2775	237	3.14	23.01	0.86	1.17	0.57
Barcode04	37,019	629	3.94	51.46	0.93	1.08	0.61
Barcode05	3310	241	3.17	23.84	0.85	1.17	0.58
Barcode06	2485	223	3.53	34.00	0.93	1.08	0.65
Barcode07	11,265	365	3.31	27.40	0.82	1.22	0.56
Barcode08	13,438	540	3.91	49.80	0.92	1.09	0.64

**Table 2:** Viral alpha diversity.

Sample Pools	Total Counts	Richness	Shannon Diversity Index (H)	Effective N Species	Simpson’s Index (D)	Inverse of D	Pielou Evenness
P1	12,788	66	0.86	2.37	0.46	2.18	0.21
P2	14,248	44	0.85	2.34	0.51	1.95	0.22

## Data Availability

The data that support the findings of this study are available from the corresponding author upon reasonable request.

## Supplementary Materials

The following supporting information can be downloaded at: https://www.mdpi.com/article/10.3390/idr15050054/s1, Figure S1: Phylogenetic diagram for each respective barcode; Figure S2: Krona visualizations illustrating the composition and distribution of taxa in the microbiome of each representative barcode; Table S1: Abundance of species corresponding to each respective barcode.

## References

[1] Kurnia N., Kaitana Y., Salaki C.L., Mandey L.C., Tuda J.S.B., Tallei T.E. (2022). Study of Dengue Virus Transovarial Transmission in *Aedes* spp. in Ternate City Using Streptavidin-Biotin-Peroxidase Complex Immunohistochemistry. Infect. Dis. Rep..

[2] Mubbashir H., Munir S., Kashif R., Nawaz H.B., Abdul B., Baharullah K. (2018). Characterization of Dengue Virus in *Aedes aegypti* and *Aedes albopictus* spp. of Mosquitoes: A Study in Khyber Pakhtunkhwa, Pakistan. Mol. Biol. Res. Commun..

[3] Trojánek M., Grebenyuk V., Manďáková Z., Sojková N., Zelená H., Roháčová H., Stejskal F. (2023). Epidemiology of Dengue, Chikungunya and Zika Virus Infections in Travellers: A 16-Year Retrospective Descriptive Study at a Tertiary Care Centre in Prague, Czech Republic. PLoS ONE.

[4] Powell J.R. (2018). Mosquito-Borne Human Viral Diseases: Why Aedes Aegypti?. Am. J. Trop. Med. Hyg..

[5] Shi H., Yu X., Cheng G. (2023). Impact of the Microbiome on Mosquito-Borne Diseases. Protein Cell.

[6] Leach C.B., Hoeting J.A., Pepin K.M., Eiras A.E., Hooten M.B., Webb C.T. (2020). Linking Mosquito Surveillance to Dengue Fever through Bayesian Mechanistic Modeling. PLoS Negl. Trop. Dis..

[7] Caragata E.P., Tikhe C.V., Dimopoulos G. (2019). Curious Entanglements: Interactions between Mosquitoes, Their Microbiota, and Arboviruses. Curr. Opin. Virol..

[8] Rückert C., Ebel G.D. (2018). How Do Virus-Mosquito Interactions Lead to Viral Emergence?. Trends Parasitol..

[9] Siriphanitchakorn T., Kini R.M., Ooi E.E., Choy M.M. (2021). Revisiting Dengue Virus-Mosquito Interactions: Molecular Insights into Viral Fitness. J. Gen. Virol..

[10] Nanfack-Minkeu F., Mitri C., Bischoff E., Belda E., Casademont I., Vernick K.D. (2019). Interaction of RNA Viruses of the Natural Virome with the African Malaria Vector, Anopheles Coluzzii. Sci. Rep..

[11] Cottis S., Blisnick A.A., Failloux A.-B., Vernick K.D. (2023). Determinants of Chikungunya and O’Nyong-Nyong Virus Specificity for Infection of Aedes and Anopheles Mosquito Vectors. Viruses.

[12] Zhou T.-F., Lai Z.-T., Liu S., Zhou J.-Y., Liu Y., Wu Y., Xu Y., Wu K., Gu J.-B., Cheng G. (2021). Susceptibility and Interactions between Aedes Mosquitoes and Zika Viruses. Insect Sci..

[13] Kerkhof L.J. (2021). Is Oxford Nanopore Sequencing Ready for Analyzing Complex Microbiomes?. FEMS Microbiol. Ecol..

[14] Tyler A.D., Mataseje L., Urfano C.J., Schmidt L., Antonation K.S., Mulvey M.R., Corbett C.R. (2018). Evaluation of Oxford Nanopore’s MinION Sequencing Device for Microbial Whole Genome Sequencing Applications. Sci. Rep..

[15] Lu J., Rincon N., Wood D.E., Breitwieser F.P., Pockrandt C., Langmead B., Salzberg S.L., Steinegger M. (2022). Metagenome Analysis Using the Kraken Software Suite. Nat. Protoc..

[16] Ondov B.D., Bergman N.H., Phillippy A.M. (2011). Interactive Metagenomic Visualization in a Web Browser. BMC Bioinform..

[17] Shen W., Ren H. (2021). TaxonKit: A Practical and Efficient NCBI Taxonomy Toolkit. J. Genet. Genom..

[18] Pérez-Cobas A.E., Gomez-Valero L., Buchrieser C. (2020). Metagenomic Approaches in Microbial Ecology: An Update on Whole-Genome and Marker Gene Sequencing Analyses. Microb. Genom..

[19] Yadav K.K., Bora A., Datta S., Chandel K., Gogoi H.K., Prasad G.B.K.S., Veer V. (2015). Molecular Characterization of Midgut Microbiota of Aedes Albopictus and Aedes Aegypti from Arunachal Pradesh, India. Parasit. Vectors.

[20] Arévalo-Cortés A., Mejia-Jaramillo A.M., Granada Y., Coatsworth H., Lowenberger C., Triana-Chavez O. (2020). The Midgut Microbiota of Colombian Aedes Aegypti Populations with Different Levels of Resistance to the Insecticide Lambda-Cyhalothrin. Insects.

[21] Guégan M., Martin E., Valiente Moro C. (2020). Comparative Analysis of the Bacterial and Fungal Communities in the Gut and the Crop of Aedes Albopictus Mosquitoes: A Preliminary Study. Pathogens.

[22] Tawidian P., Coon K.L., Jumpponen A., Cohnstaedt L.W., Michel K. (2021). Host-Environment Interplay Shapes Fungal Diversity in Mosquitoes. mSphere.

[23] Araújo J.P.M., Hughes D.P., Lovett B., St. Leger R.J. (2016). Chapter One—Diversity of Entomopathogenic Fungi: Which Groups Conquered the Insect Body?. Genetics and Molecular Biology of Entomopathogenic Fungi.

[24] Fatimawali, Kepel B.J., Gani M.A., Tallei T.E. (2020). Comparison of Bacterial Community Structure and Diversity in Traditional Gold Mining Waste Disposal Site and Rice Field by Using a Metabarcoding Approach. Int. J. Microbiol..

[25] Schwartz M.W., Brigham C.A., Hoeksema J.D., Lyons K.G., Mills M.H., van Mantgem P.J. (2000). Linking Biodiversity to Ecosystem Function: Implications for Conservation Ecology. Oecologia.

[26] Tallei T.E., Fatimawali, Pelealu J.J. (2019). The Data on Metagenomic Profile of Bacterial Diversity Changes in the Different Concentration of Fermented Romaine Lettuce Brine. Data Brief.

[27] Cao Y., Hawkins C.P. (2019). Weighting Effective Number of Species Measures by Abundance Weakens Detection of Diversity Responses. J. Appl. Ecol..

[28] Tallei V.R., Saroyo, Tallei T.E. (2016). Diversity of Recorded Wild Mammals in Mount Tumpa Forest Park, North Sulawesi, Indonesia. J. Plant Sci..

[29] Niode N.J., Adji A., Rimbing J., Tulung M., Tallei T.E. (2021). Composition and Diversity of Bacteria from Giant Asian Honeybee Apis Dorsata Gut. Biodiversitas.

[30] Pielou E.C. (1966). The Measurement of Diversity in Different Types of Biological Collections. J. Theor. Biol..

[31] Liu Q., Cui F., Liu X., Fu Y., Fang W., Kang X., Lu H., Li S., Liu B., Guo W. (2023). Association of Virome Dynamics with Mosquito Species and Environmental Factors. Microbiome.

[32] Kuhn J.H., Adkins S., Agwanda B.R., Al Kubrusli R., Alkhovsky S.V., Amarasinghe G.K., Avšič-Županc T., Ayllón M.A., Bahl J., Balkema-Buschmann A. (2021). 2021 Taxonomic Update of Phylum Negarnaviricota (Riboviria: Orthornavirae), Including the Large Orders Bunyavirales and Mononegavirales. Arch. Virol..

[33] Kanojia A., Sharma M., Shiraz R., Tripathi S. (2022). Flavivirus-Host Interaction Landscape Visualized through Genome-Wide CRISPR Screens. Viruses.

[34] Teo D., Ng L.C., Lam S. (2009). Is Dengue a Threat to the Blood Supply?. Transfus. Med..

[35] Duong V., Lambrechts L., Paul R.E., Ly S., Lay R.S., Long K.C., Huy R., Tarantola A., Scott T.W., Sakuntabhai A. (2015). Asymptomatic Humans Transmit Dengue Virus to Mosquitoes. Proc. Natl. Acad. Sci. USA.

[36] Brackney D.E., LaReau J.C., Smith R.C. (2021). Frequency Matters: How Successive Feeding Episodes by Blood-Feeding Insect Vectors Influences Disease Transmission. PLoS Pathog..

