# Supplementary material for: Metagenomic Insight into the Microbiome and Virome Associated with Aedes aegypti Mosquitoes in Manado (North Sulawesi, Indonesia)
Source: Infect Dis Rep. 2023 Sep 11;15(5):549–63. doi: 10.3390/idr15050054 (PMC10514871; doi:10.3390/idr15050054)
Supplement: Supplementary file 1 [file idr-15-00054-s001.zip › Supplementary Figure S2.pdf]

Supplementary Figure S2: Krona visualizations illustrating the composition and distribution of taxa in the microbiome of each representative Barcode.

Barcode03

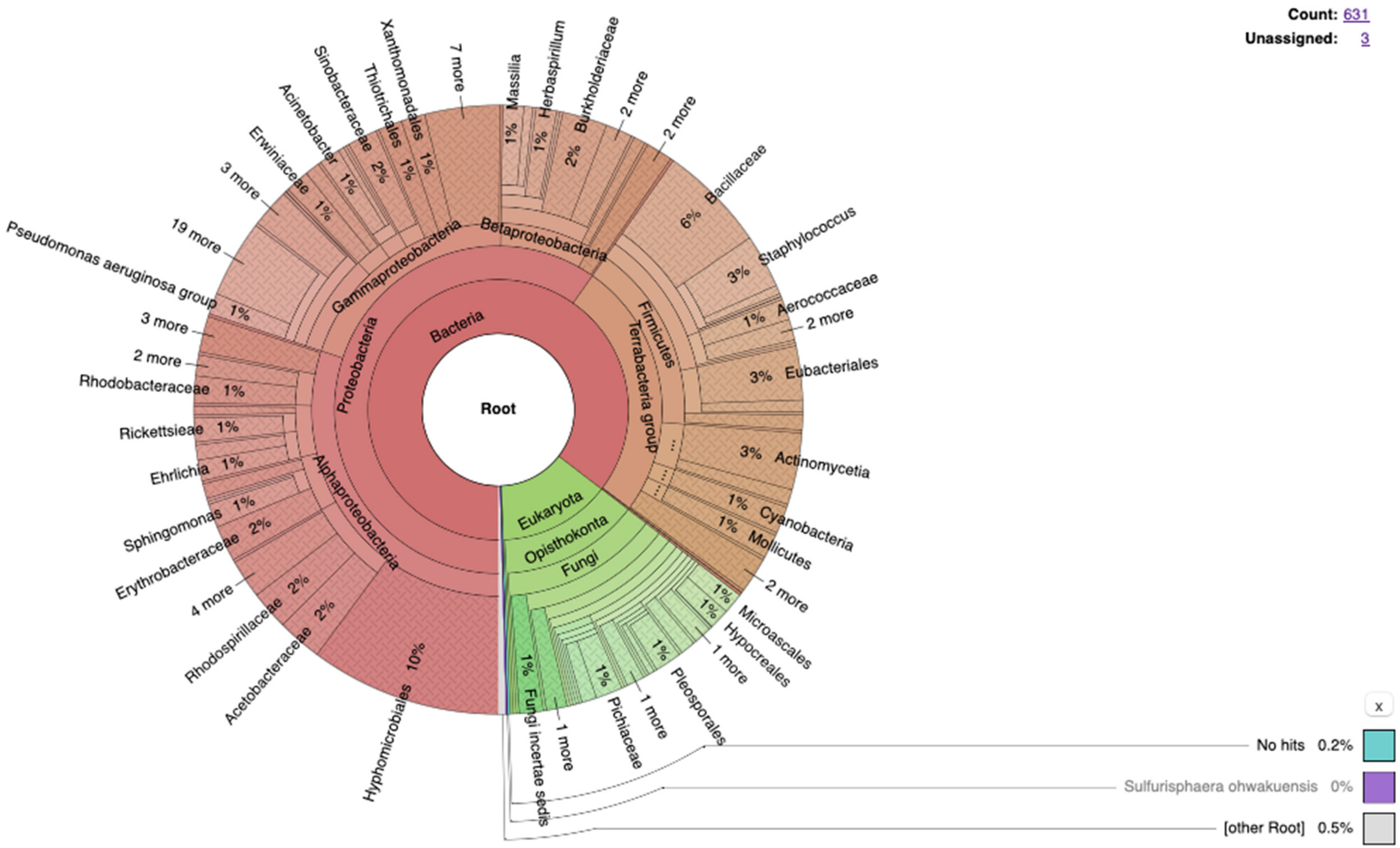

Barcode04

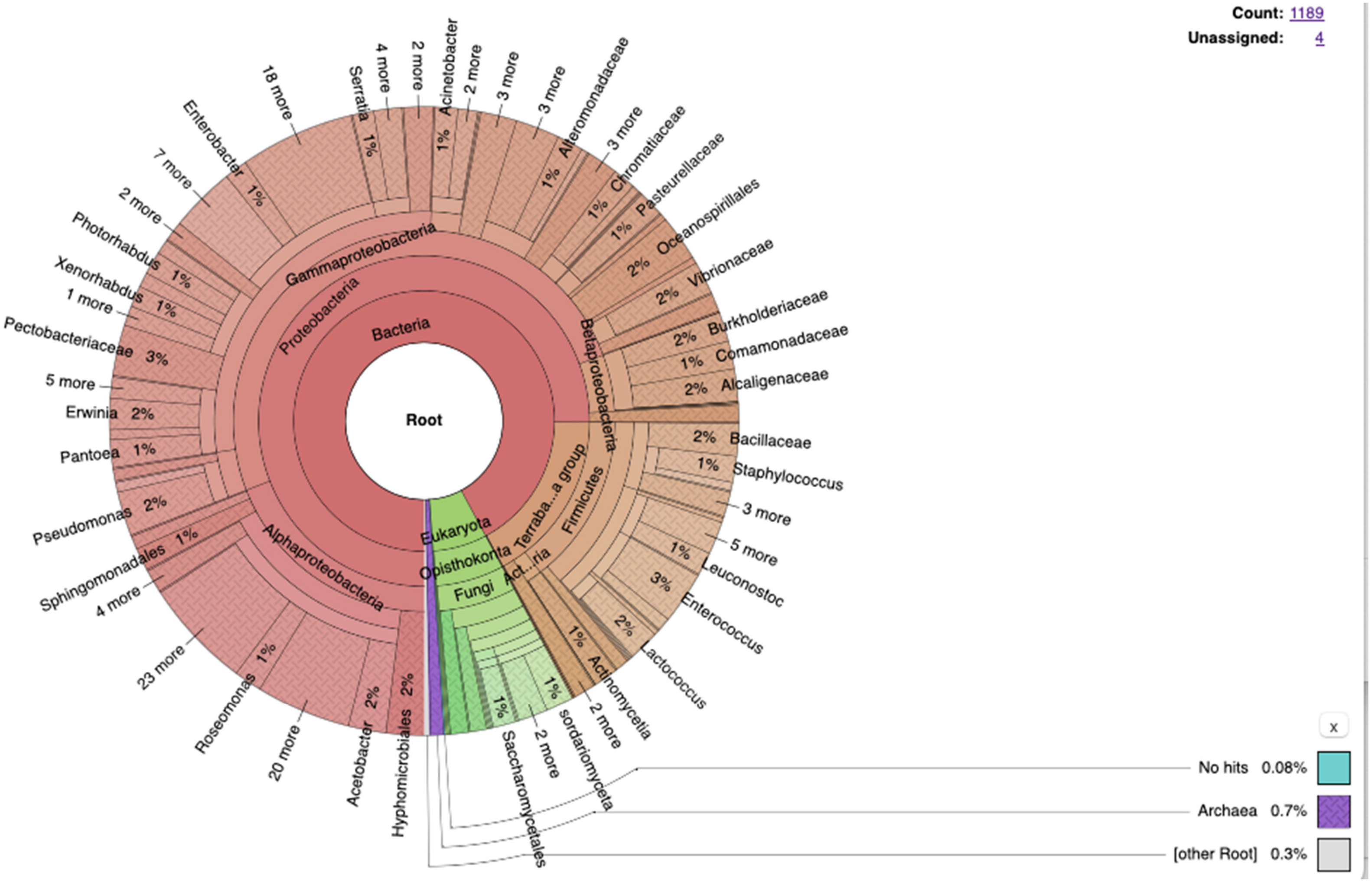

Count: 1189
Unassigned: 4
Root
Bacteria
Proteobacteria
Gammaproteobacteria
Betaproteobacteria
Alphaproteobacteria
Terraba...a group
Firmicutes
Act...ria
Eukaryota
Opisthokonta
Fungi
Enterobacter 1%
18 more
Serratia 1%
4 more
2 more
Acinetobacter 1%
2 more
3 more
3 more
Alteromonadaceae 1%
3 more
Chromatiaceae 1%
Pasteurellaceae 1%
Oceanospirillales 2%
Vibrionaceae 2%
Burkholderiaceae 2%
Comamonadaceae 1%
Alcaligenaceae 2%
Bacillaceae 2%
Staphylococcus 1%
3 more
5 more
Leuconostoc 1%
Enterococcus 3%
Lactococcus 2%
Actinomycetia 1%
2 more
sordariomyceta 1%
2 more
Saccharomycetales 1%
Hyphomicrobiales 2%
Acetobacter 2%
20 more
Roseomonas 1%
23 more
4 more
Sphingomonadales 1%
Pseudomonas 2%
Pantoea 1%
Erwinia 2%
5 more
Pectobacteriaceae 3%
1 more
Xenorhabdus 1%
Photorhabdus 1%
2 more
7 more
x
No hits 0.08%
Archaea 0.7%
[other Root] 0.3%

Barcode05

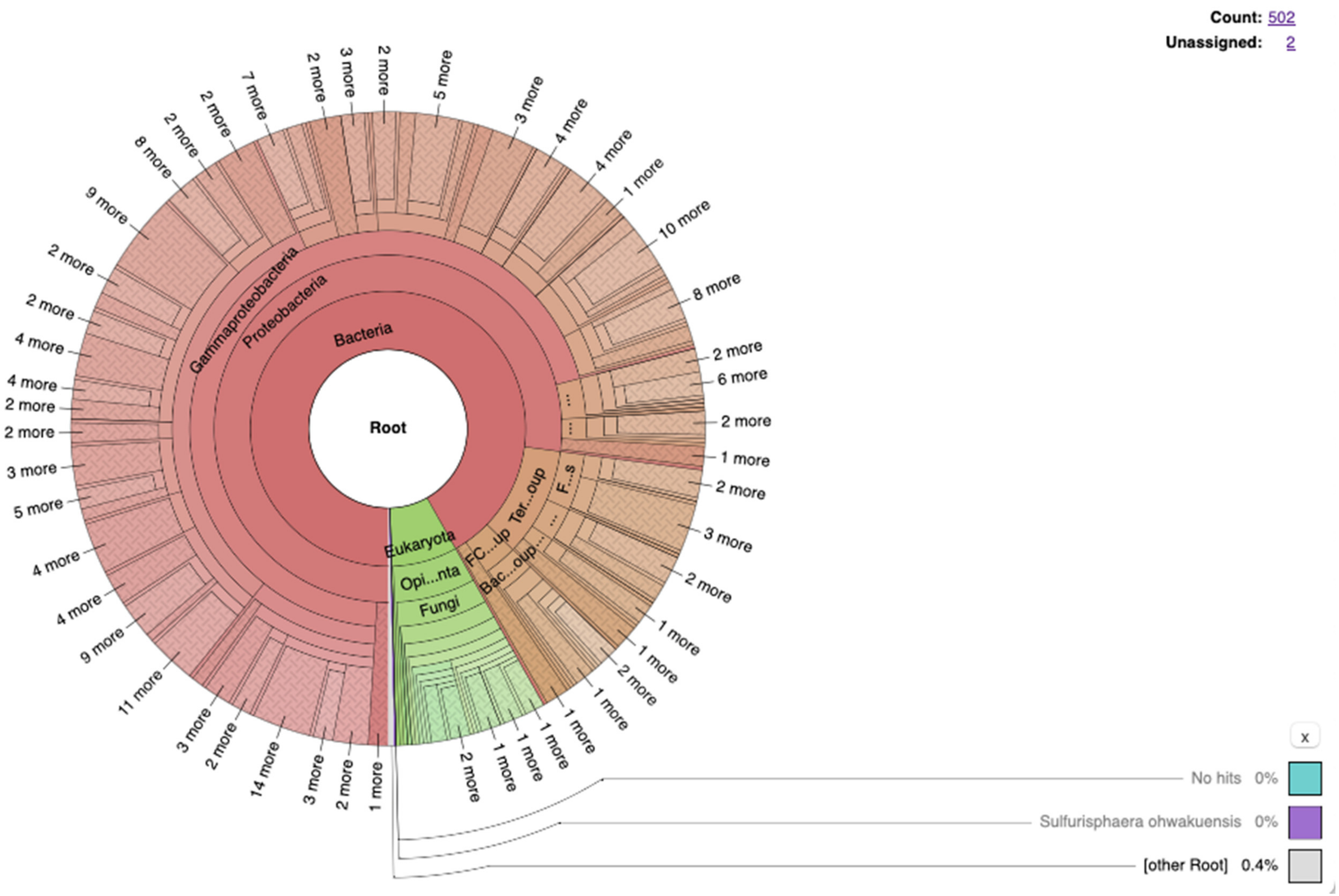
Count: 502
Unassigned: 2
Root
Bacteria
Proteobacteria
Gammaproteobacteria
Eukaryota
Opi...nta
Fungi
FC...up
Ter...oup
Bac...oup
F...s
No hits 0%
Sulfurisphaera ohwakuensis 0%
[other Root] 0.4%

Barcode06

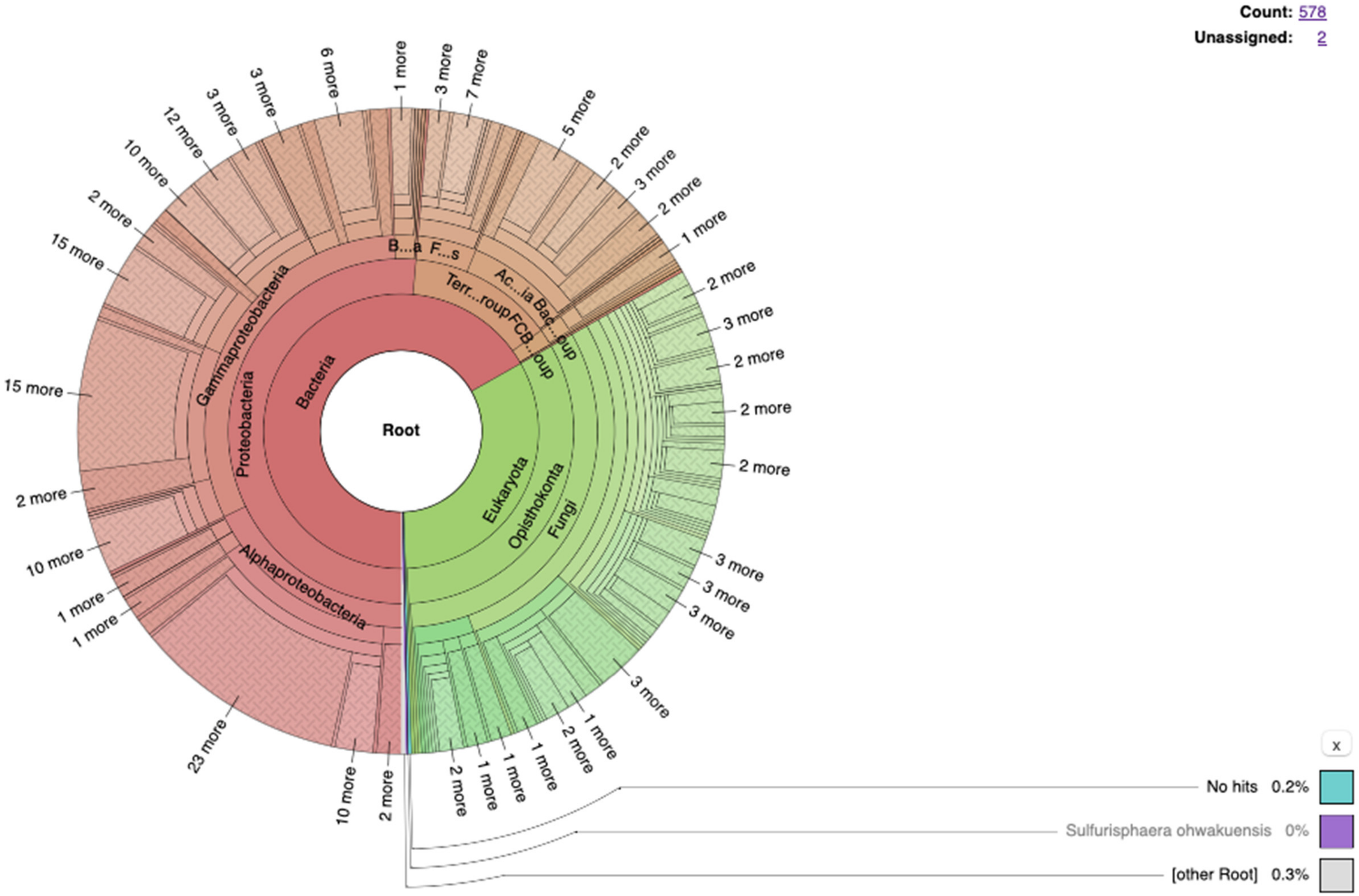
Count: 578
Unassigned: 2
Root
Bacteria
Proteobacteria
Gammaproteobacteria
Alphaproteobacteria
B...a
F...s
Terr...roup
FCB...oup
Ac...ia
Bac...oup
Eukaryota
Opisthokonta
Fungi
No hits 0.2%
Sulfurisphaera ohwakuensis 0%
[other Root] 0.3%

Barcode07

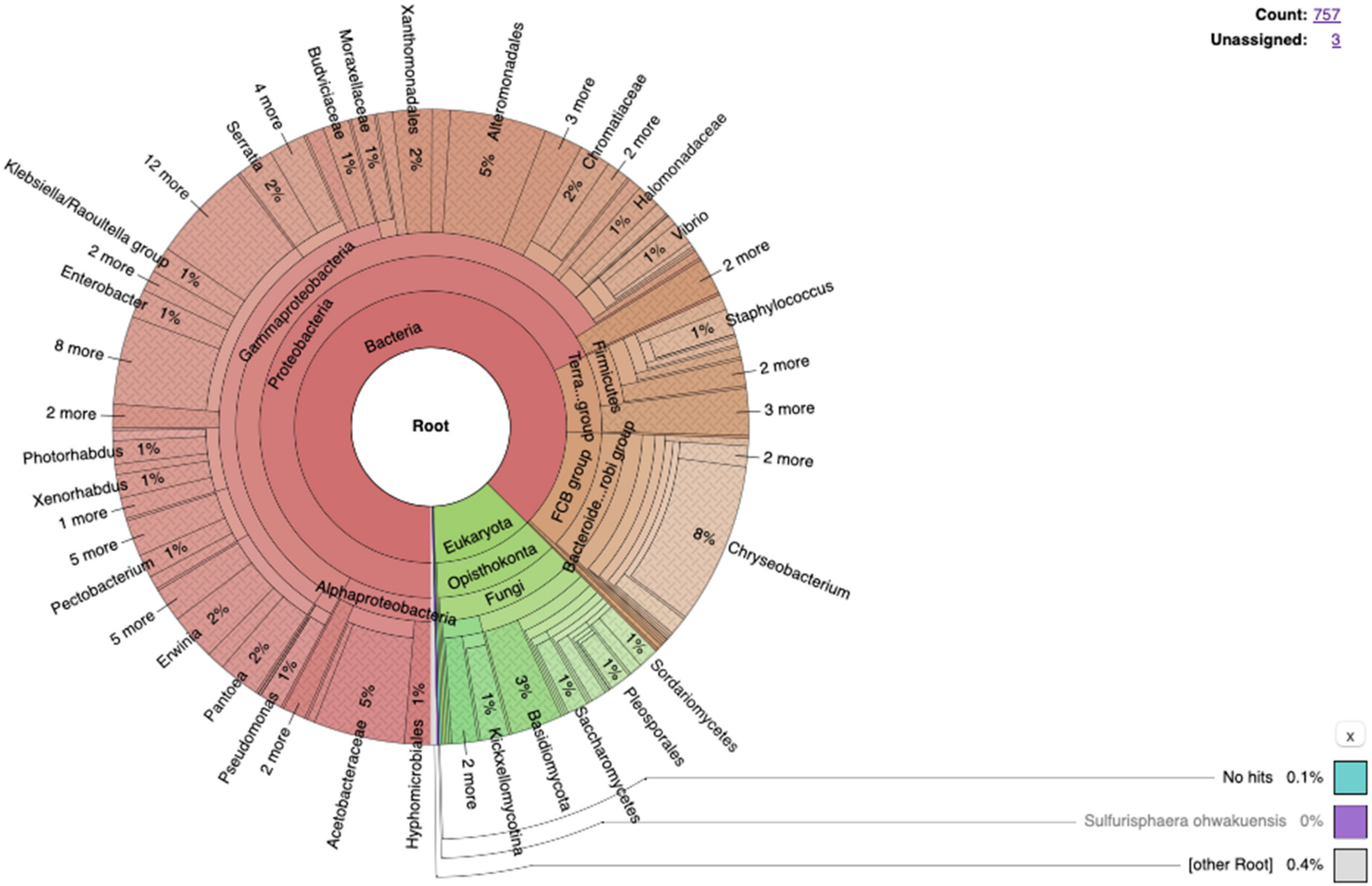
Count: 757
Unassigned: 3
Root
Bacteria
Proteobacteria
Gammaproteobacteria
Alphaproteobacteria
Eukaryota
Opisthokonta
Fungi
FCB group
Bacteroide...robi group
Terra...group
Firmicutes
Xanthomonadales 2%
Alteromonadales 5%
3 more
Chromatiaceae 2%
2 more
Halomonadaceae 1%
Vibrio 1%
2 more
Staphylococcus 1%
2 more
3 more
2 more
Chryseobacterium 8%
Sordariomycetes 1%
Pleosporales 1%
Saccharomycetes 1%
Basidiomycota 3%
Kickxellomycotina 1%
2 more
Hyphomicrobiales 1%
Acetobacteraceae 5%
2 more
Pseudomonas 1%
Pantoea 2%
Erwinia 2%
5 more
Pectobacterium 1%
5 more
1 more
Xenorhabdus 1%
Photorhabdus 1%
2 more
8 more
Enterobacter 1%
2 more
Klebsiella/Raoultella group 1%
12 more
Serratia 2%
4 more
Budviciaceae 1%
Moraxellaceae 1%
x
No hits 0.1%
Sulfurisphaera ohwakuensis 0%
[other Root] 0.4%

Barcode08

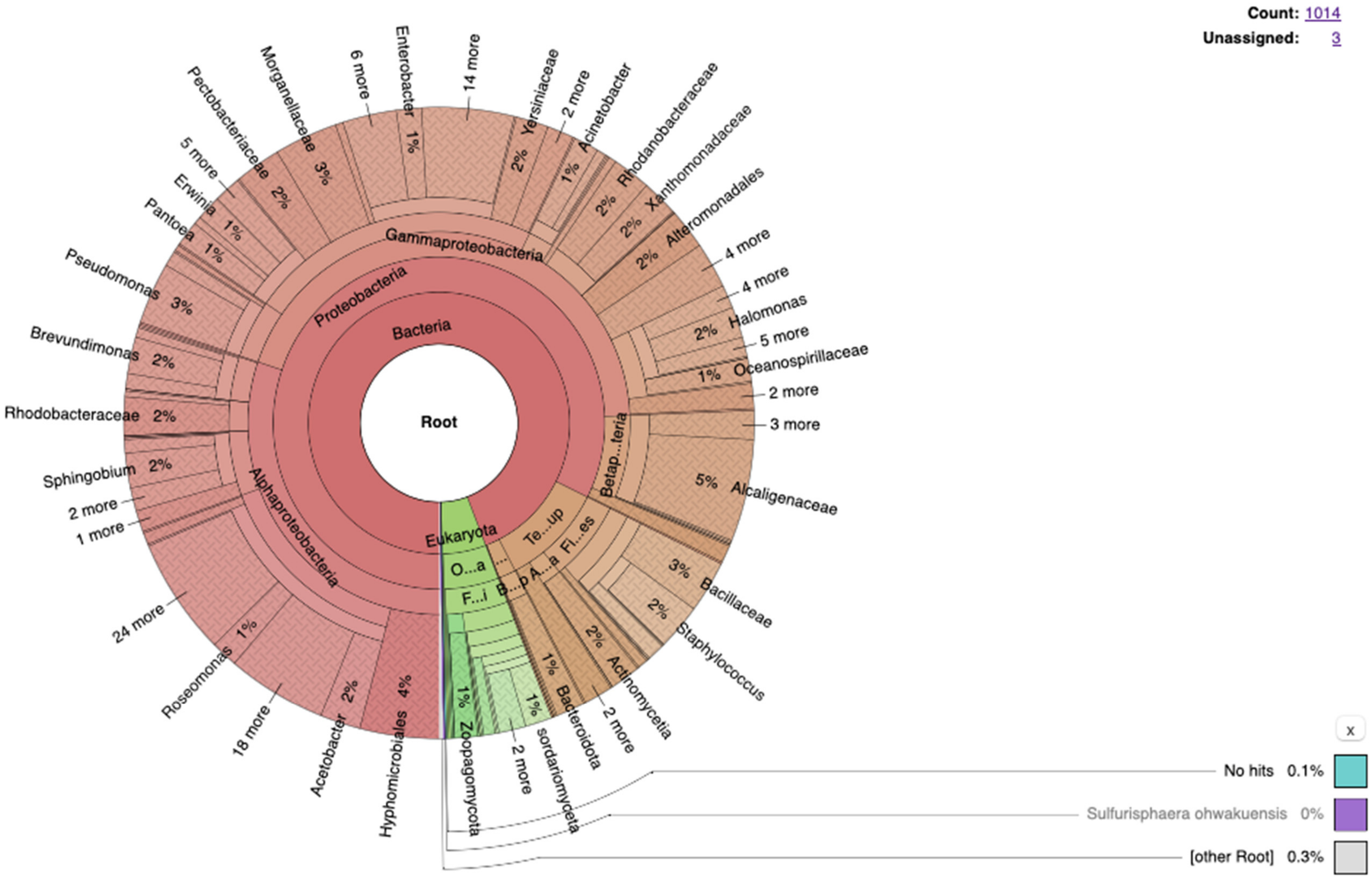

Count: 1014
Unassigned: 3
Root
Bacteria
Proteobacteria
Gammaproteobacteria
Alphaproteobacteria
Betap...teria
Eukaryota
Te...up
Fi...es
O...a
F...i
B...p
A...a
Pectobacteriaceae 2%
Morganellaceae 3%
6 more
Enterobacter 1%
14 more
Yersiniaceae 2%
2 more
Acinetobacter 1%
Rhodanobacteraceae 2%
Xanthomonadaceae 2%
Alteromonadales 2%
4 more
4 more
Halomonas 2%
5 more
Oceanospirillaceae 1%
2 more
3 more
Alcaligenaceae 5%
Bacillaceae 3%
Staphylococcus 2%
Actinomycetia 2%
2 more
Bacteroidota 1%
sordariomyceta 1%
2 more
Zoopagomycota 1%
Hyphomicrobiales 4%
Acetobacter 2%
18 more
Roseomonas 1%
24 more
1 more
2 more
Sphingobium 2%
Rhodobacteraceae 2%
Brevundimonas 2%
Pseudomonas 3%
Pantoea 1%
Erwinia 1%
5 more
x
No hits 0.1%
Sulfurisphaera ohwakuensis 0%
[other Root] 0.3%
