# Supplementary material for: Metagenomic Insight into the Microbiome and Virome Associated with Aedes aegypti Mosquitoes in Manado (North Sulawesi, Indonesia)
Source: Infect Dis Rep. 2023 Sep 11;15(5):549–63. doi: 10.3390/idr15050054 (PMC10514871; doi:10.3390/idr15050054)
Supplement: Supplementary file 1 [file idr-15-00054-s001.zip › Supplementary Table S1.pdf]

Supplementary Table S1: Abundance of species corresponding to each respective barcode.

	Species	Abundance of Each Species Within the Respective Barcode.					
		**Barcode03**	**Barcode04**	**Barcode05**	**Barcode06**	**Barcode07**	**Barcode08**
Fungi	*Brettanomyces* sp.	44					
	Brettanomyces acidodurans				139		
	Brettanomyces bruxellensis				42		
	Brettanomyces custersianus				31		
Bacteria	*Acetobaccter oryzoeni*		537		41		
	Acinetobacter indicus				140		
	Aeromonas hydrophila			99			
	Aeromonas veronii			48			
	Anaplasma phagocytophilum	318					
	Asaia bogorensis		8,809				
	Asaia krungthepensis		591		347		
	Asaia lannensis				49		
	Asaia prunellae				25		
	Chronobacter muytjensii			34			
	Citrobacter koseri		717	273		671	
	Cutibacterium acne	129					
	Dermacoccus barathri			29			
	Ehrlichia muris	58					
	Ehrlichia minasensi	266					
	Ehrlichia chaffensis	39					
	Enterobacter chuandaensis			321			
	Enterococcus sulfureus		371				
	Erwinia piriflorinigrans		926	91		4,609	174
	Franconibacter sp.						147
	Franconibacter helveticus			60		185	
	Franconibacter daqui			48		179	
	Geminococcus roseus				46		
	Haemophilus parainfluenzae				214		
	Herbaspirillum frisingense	60					

	Klebsiella pneumoniae		548			136	
	Kluyvera ascorbata		849	154		475	228
	Komagataeibacter europaeus						144
	Lactococcus lactis		2,422				
	Moraxella osloensis				423		
	Pantoea stewartii			1,170			
	Pectobacterium peruviense					176	
	Pheylobacterium haematohilum	34					
	Proteus columbae		851				
	Proteus faecis		1,390				
	Proteus hauseri		634				
	Proteus vulgaris		621				
	Providencia stuartii		983				
	Pseudoxanthomonas kaohsiungensis				107		
	Pseudoxanthomona yeongjuensis				42		
	Rahnella inusitata		749		42	205	174
	Rickettsia honei	32					
	Staphylococcus sp.						149
	Staphylococcus massiliens	38					
	Staphylococcus capitis	71					
	Staphylococus hominis				121		
	Spiroplasma ixodetis	944					
	Tatumella terea			55		182	
	Yersenia canariae					305	142
	Zhongshania aliphaticivorans	63					
	Zymobacter palmae						388
