# Supplementary figures and images for: Metagenomic Insight into the Microbiome and Virome Associated with Aedes aegypti Mosquitoes in Manado (North Sulawesi, Indonesia)
Source: Infect Dis Rep. 2023 Sep 11;15(5):549–63. doi: 10.3390/idr15050054 (PMC10514871; doi:10.3390/idr15050054)

Barcode03

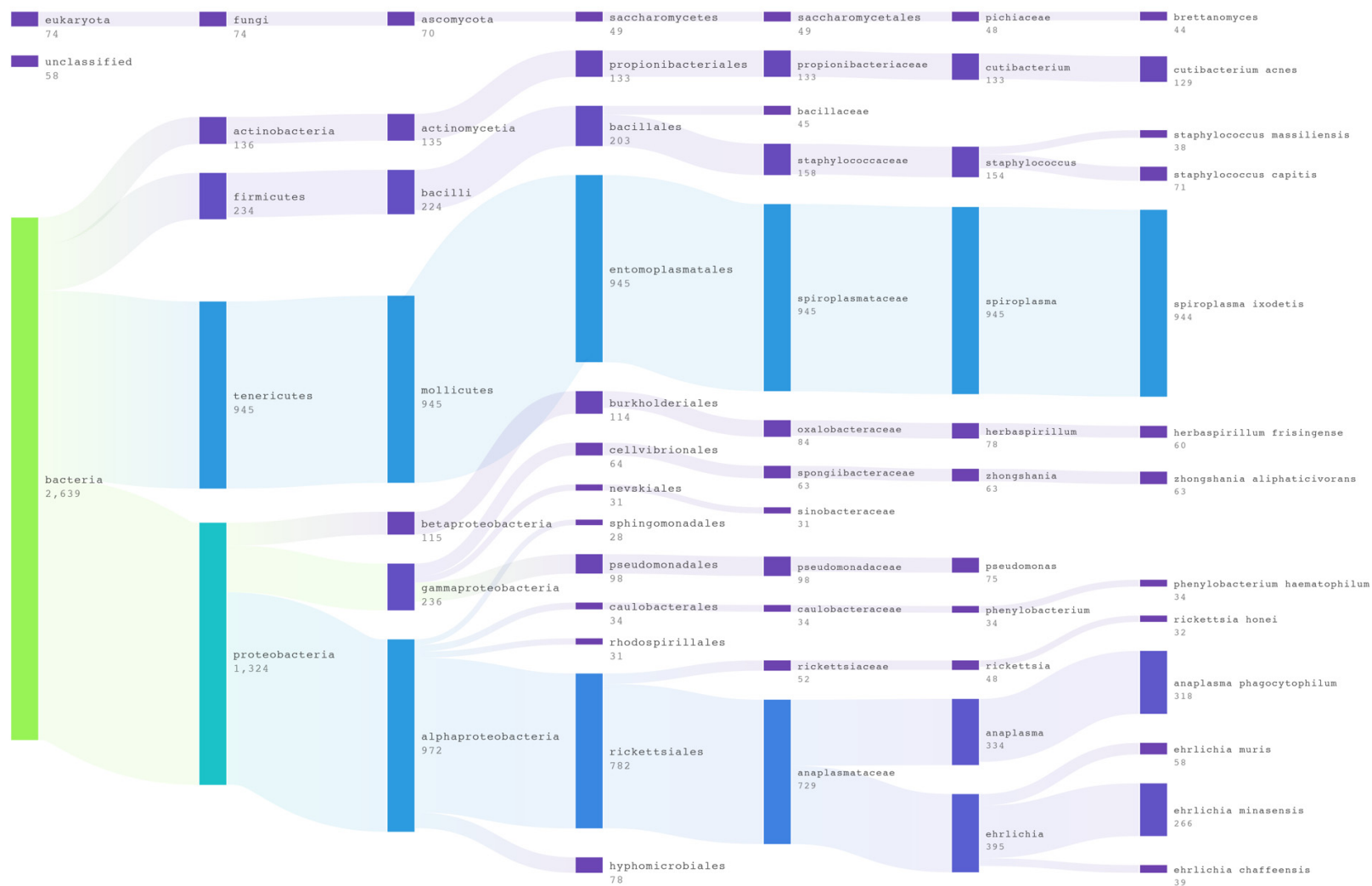

Barcode04

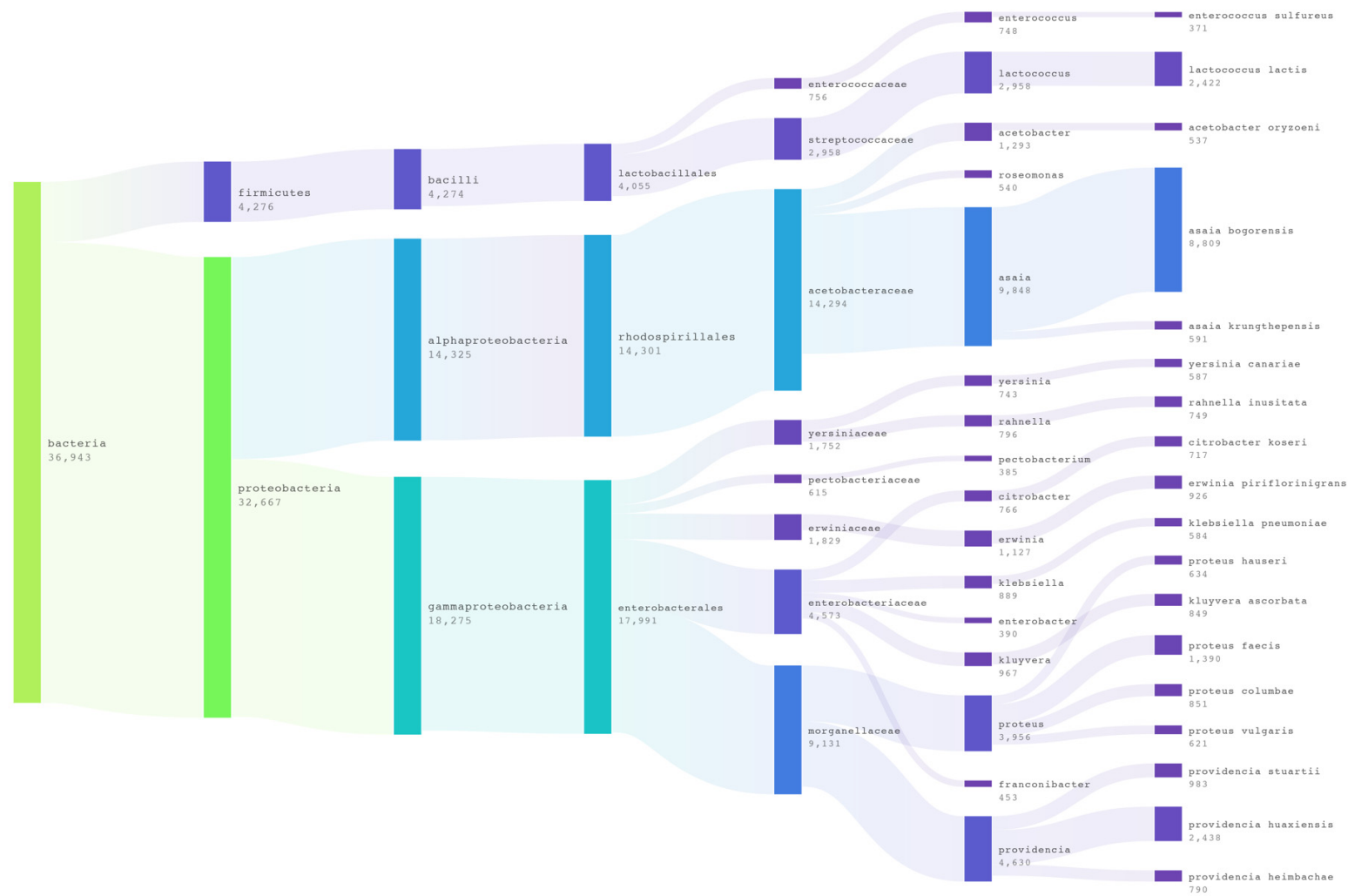

Barcode05

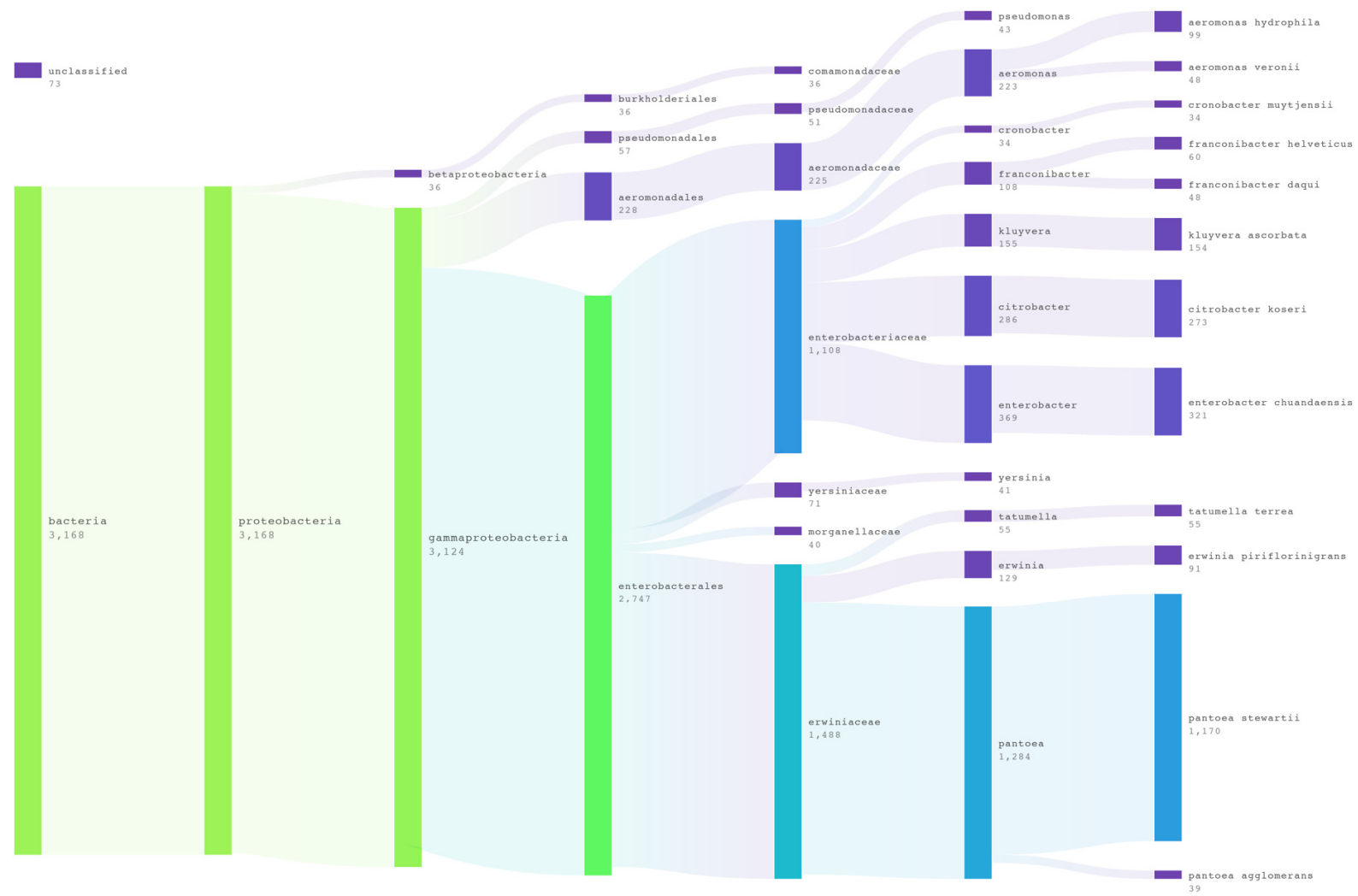

## Barcode06

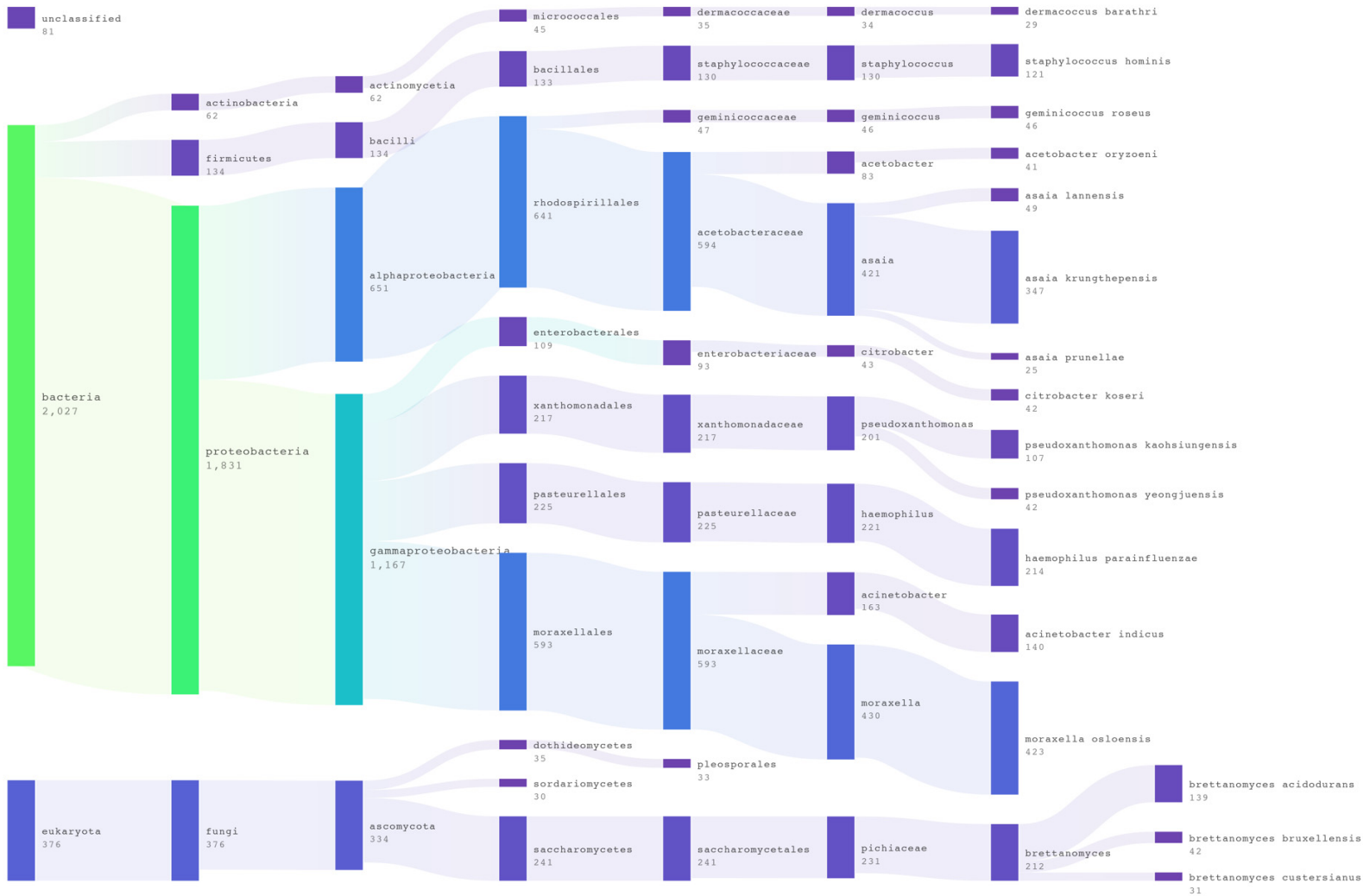

Barcode07

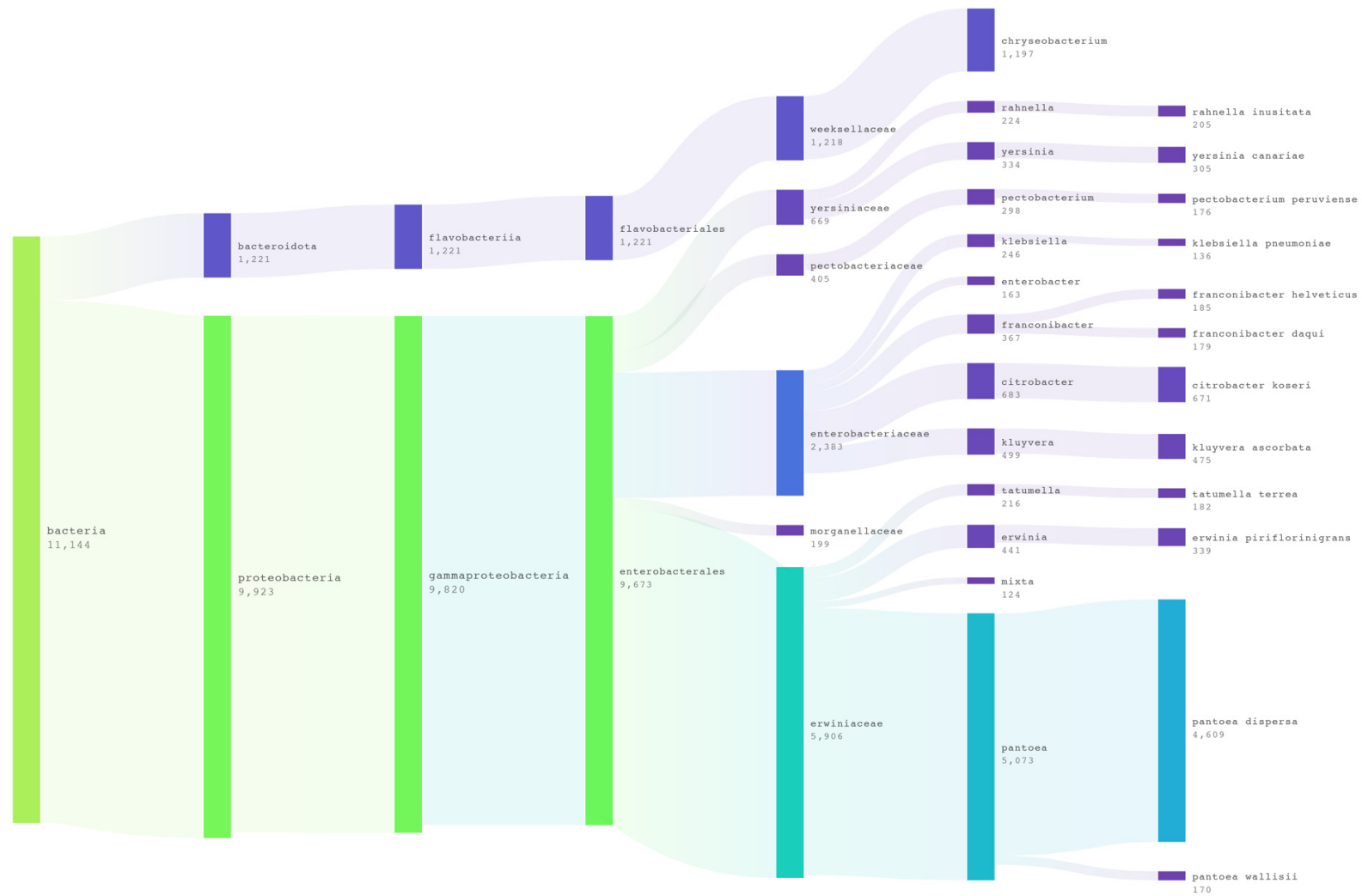

Barcode08

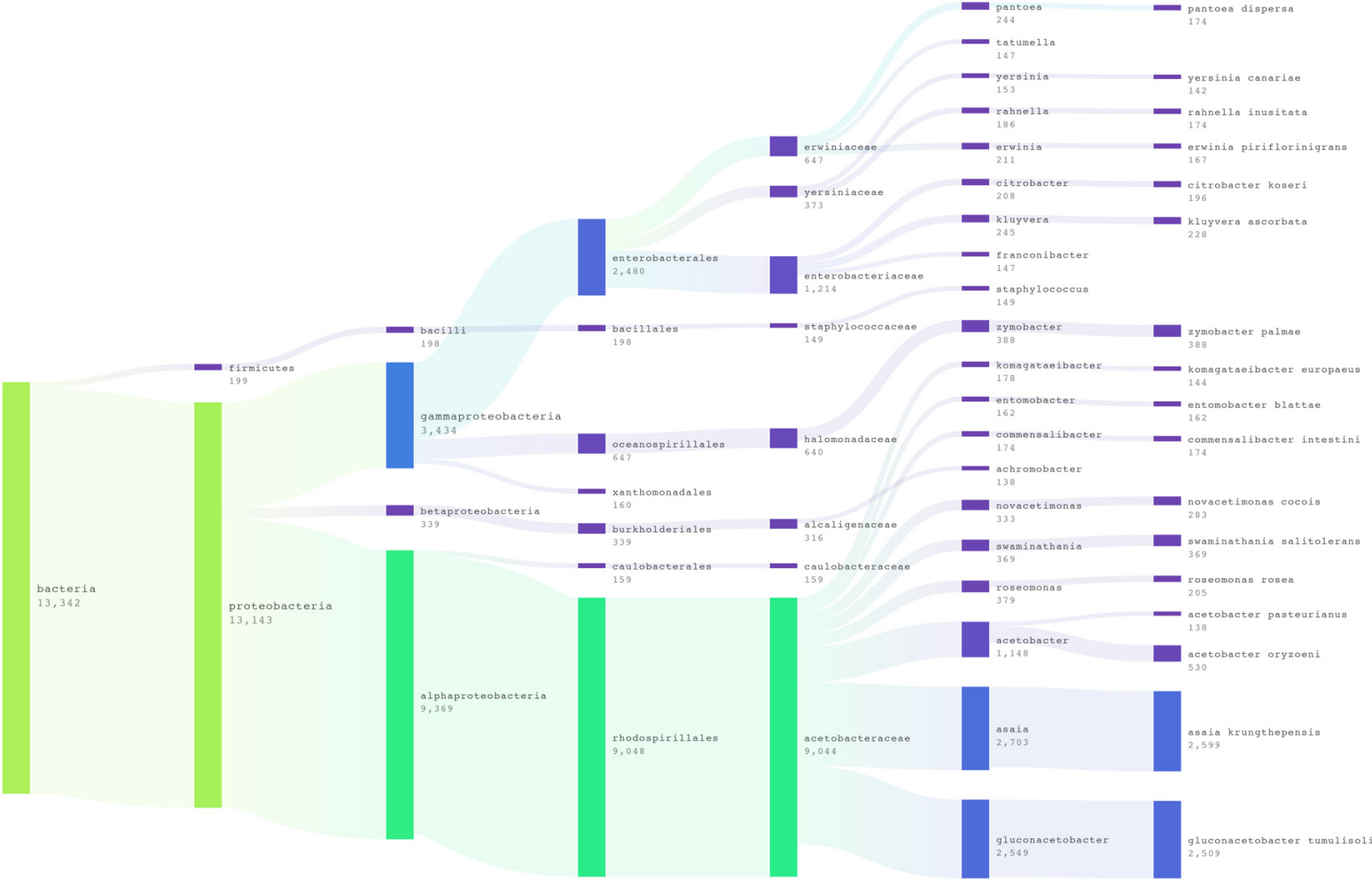

Supplement: Supplementary file 1 [file idr-15-00054-s001.zip › Supplementary Figure S1.pdf]
